# Discovery of α-(1→6)-linked mannan structures resembling yeast *N*-glycan outer chains in *Aspergillus fumigatus* mycelium

**DOI:** 10.1128/msphere.00100-24

**Published:** 2024-04-23

**Authors:** Chihiro Kadooka, Yutaka Tanaka, Rintaro Kishida, Daisuke Hira, Takuji Oka

**Affiliations:** 1 Department of Biotechnology and Life Sciences, Faculty of Biotechnology and Life Sciences, Sojo University, Kumamoto, Japan; 2 Division of Infection and Host Defense, Tohoku Medical and Pharmaceutical University, Sendai, Japan; University of Georgia, Athens, Georgia, USA

**Keywords:** mannosyltransferase, cell wall, outer chain, mannan, *Aspergillus fumigatus*

## Abstract

The cellular surface of the pathogenic filamentous fungus *Aspergillus fumigatus* is enveloped in a mannose layer, featuring well-established fungal-type galactomannan and *O*-mannose-type galactomannan. This study reports the discovery of cell wall component in *A. fumigatus* mycelium, which resembles *N*-glycan outer chains found in yeast. The glycosyltransferases involved in its biosynthesis in *A. fumigatus* were identified, with a focus on two key α-(1→2)-mannosyltransferases, Mnn2 and Mnn5, and two α-(1→6)-mannosyltransferases, Mnn9 and Van1. *In vitro* examination revealed the roles of recombinant Mnn2 and Mnn5 in transferring α-(1→2)-mannosyl residues. Proton nuclear magnetic resonance (^1^H-NMR) analysis of cell wall extracts from the ∆*mnn2*∆*mnn5* strain indicated the existence of an α-(1→6)-linked mannan backbone in the *A. fumigatus* mycelium, with Mnn2 and Mnn5 adding α-(1→2)-mannosyl residues to this backbone. The α-(1→6)-linked mannan backbone was absent in strains where *mnn9* or *van1* was disrupted in the parental ∆*mnn2*∆*mnn5* strain in *A. fumigatus*. Mnn9 and Van1 functioned as α-(1→6)-linked mannan polymerases in heterodimers when co-expressed in *Escherichia coli*, indicating their crucial role in biosynthesizing the α-(1→6)-linked mannan backbone. Disruptions of these mannosyltransferases did not affect fungal-type galactomannan biosynthesis. This study provides insights into the complexity of fungal cell wall architecture and a better understanding of mannan biosynthesis in *A. fumigatus*.

**IMPORTANCE:**

This study unravels the complexities of mannan biosynthesis in *A. fumigatus*, a key area for antifungal drug discovery. It reveals the presence of α-(1→6)-linked mannan structures resembling yeast N-glycan outer chains in *A. fumigatus* mycelium, offering fresh insights into the fungal cell wall’s design. Key enzymes, Mnn2, Mnn5, Mnn9, and Van1, are instrumental in this process, with Mnn2 and Mnn5 adding specific mannose residues and Mnn9 and Van1 assembling the α-(1→6)-linked mannan structures. Although fungal-type galactomannan’s presence in the cell wall is known, the existence of an α-(1→6)-linked mannan adds a new dimension to our understanding. This intricate web of mannan biosynthesis opens avenues for further exploration and enhances our understanding of fungal cell wall dynamics, paving the way for targeted drug development.

## INTRODUCTION

Mannan plays a crucial role in maintaining the cell wall structure in yeast and filamentous fungi. In *Aspergillus fumigatus*, a prominent pathogenic fungus causing invasive pulmonary aspergillosis, mannose-containing sugar chains are integral components found in *N*- and *O*-linked glycans on various proteins, glycosylphosphatidylinositol (GPI) anchor, mannosylinositol phosphorylceramides (MIPC), and fungal-type galactomannan (FTGM) (1–5). Studies from our group and others have revealed that disrupting *cmsA*/*ktr4*, an α-(1→2)-mannosyltransferase encoding gene responsible for FTGM α-core mannan synthesis, leads to abnormal colony morphology and a reduction in infectious capacity in *A. fumigatus* (6, 7). Furthermore, the double disruption of *pmt4* and *pmt1*, encoding protein *O*-mannosyltransferases, is synthetically lethal in *A. fumigatus*, emphasizing the essential role of protein *O*-mannosylation in hyphal growth through the maintenance of cell surface and secreted proteins (8). Additionally, disrupting *mnt1*, an α-(1→2)-mannosyltransferase gene responsible for decorating the second mannosyl residue to protein *O*-mannose-type galactomannan (OMGM) and elongating high mannose type *N*-glycan core chain, results in a thin cell wall and hypovirulence in *A. fumigatus* (9, 10). Therefore, comprehensively understanding mannan biosynthesis in *A. fumigatus* is crucial for advancing novel antifungal drug discovery.

In *Saccharomyces cerevisiae*, *N*-glycans attached to proteins exhibit extensive mannan structures, referred to as outer chains, consisting of up to 200 mannosyl residues (11). The biosynthesis of outer chain structures is well-studied in *S. cerevisiae*. The initial reaction is catalyzed by the specific α-(1→6)-mannosyltransferase *Sc*Och1p (12). Subsequently, the elongation of at least 10 mannosyl residues is facilitated by mannan polymerase I (M-Pol I), a heterodimeric complex of two α-(1→6)-mannosyltransferases, *Sc*Mnn9p and *Sc*Van1p, in *S. cerevisiae* (13–15). The α-(1→6)-linked mannan backbone is further extended by another mannan polymerase complex, M-Pol II, consisting of α-(1→6)-mannosyltransferases *Sc*Mnn9p, *Sc*Anp1p, *Sc*Mnn10p, *Sc*Mnn11p, and *Sc*Hoc1p in *S. cerevisiae* (16, 17). Additionally, α-(1→2)-, α-(1→3)-, and mannose-6 phosphate side chains are decorated by α-(1→2)-mannosyltransferases *Sc*Mnn2p and *Sc*Mnn5p (18), α-(1→3)-mannosyltransferase Mnn1p (19), and mannose-6 phosphate transferase *Sc*Mnn4p and *Sc*Mnn14p (20, 21) in *S. cerevisiae*. The visualization of the outer chain α-(1→6)-linked mannan core backbone in a ∆*Scmnn2* strain identified *Sc*Mnn2p as an α-(1→2)-mannosyltransferase responsible for the initial attachment of α-(1→2)-mannosyl residues in the side chain of *N*-glycan outer chain (18, 22) in *S. cerevisiae*. Subsequently, *Sc*Mnn5p adds a second α-(1→2)-mannosyl residue to the side chain in *S. cerevisiae* (18). Finally, the *N*-glycan outer chain structures are capped in *S. cerevisiae* through the addition of mannose 6-phosphate and α-(1→3)-mannosyl residues onto (1→2)-mannosyl residues, dependent by *Sc*Mnn4p, *Sc*Mnn14p, and *Sc*Mnn1p (20, 21, 23).

The initiating α-(1→6)-mannosyltransferase, *Sc*Och1p, serves as a pivotal enzyme in the biosynthesis of outer chain structures in *S. cerevisiae* (12). Analyses of *Sc*Och1p orthologs in various models and pathogenic fungi have been conducted (12, 24–28). In the pathogenic yeast *Candida albicans*, disruption of *Caoch1* strongly induces growth defects and reduces infection survival rates (24). Similarly, the *och-1* disruptant exhibited severe growth defects and abnormal conidial formations in *Neurospora crassa*, suggesting the presence of *N*-glycan outer chain structures not only in yeast but also in filamentous fungi (25). Contrastingly, in *A. fumigatus*, disruption of the *och1* gene resulted in only growth defects and abnormal conidia formations under calcium stress conditions (26). A quadruple disruptant of four *Sc*Och1p ortholog genes (*och1-1*, *och1-2*, *och1-3*, and *och1-4*) does not exhibit any phenotypes in *A. fumigatus*, suggesting a potential divergence in mannan biosynthesis pathways between *A. fumigatus*, other filamentous fungi, and yeast (27). Henry et al. described the *A. fumigatus* undecuple Δ*11* strain (*mnn9*, *van1*, *anpA*, *mnn10*, *mnn11*, *mnn2*, *mnn5*, *och1-1*, *och1-2*, *och1-3*, and *och1-4* genes multiple-deletion mutant), which showed a growth defect and reduced cell wall thickness and alkali-soluble mannan content, indicating the possible roles of these mannosyltransferases in biosynthesis of specific mannan structures (28). Du et al. reported the requirement of the *Sc*Mnn9p ortholog for cell wall integrity through cell wall mannan and/or mannoprotein biosynthesis in *A. fumigatus* (29). Additionally, a recent study by Liu et al. revealed an unknown glycan structure named G3Man in *A. fumigatus*, specifically present in the conidia (30). The GT62 and GT32 genes (including *mnn9*, *van1*, and *anpA* and *och1-1*, *och1-2*, *och1-3*, and *och1-4*, respectively) are implicated in the synthesis of G3Man in *A. fumigatus* (30). Unlike the yeast outer chain, G3Man lacks α-(1→2)-mannosyl side chains on the α-(1→6)-linked mannan backbone but features side chains of galactose, glucose, and *N*-acetylglucosamine (30). However, the functions of Mnn2 and Mnn5 homologs in filamentous fungi and the presence and importance of the outer chain in mycelia remain unclear.

In our study, we specifically focused on two putative α-(1→2)-mannosyltransferase, Mnn2 and Mnn5, in *A. fumigatus*. Recombinant *Af*Mnn2 and *Af*Mnn5 had α-(1→2)-mannosyltransferase activity *in vitro*. Although ∆*mnn2* or ∆*mnn5* mutants showed no discernible phenotype, the ∆*mnn2*∆*mnn5* double mutant exhibited a growth defect and abnormal conidial formations in *A. fumigatus*. Proton nuclear magnetic resonance (^1^H-NMR) analysis of the total mannan fraction from the ∆*mnn2*∆*mnn5* mutant provided compelling evidence for the presence of α-(1→6)-linked mannan backbone in *A. fumigatus* mycelia. Furthermore, Mnn9 and Van1 were identified as key enzymes in the biosynthesis of this mannan backbone in *A. fumigatus*. In our *in vitro* experiments, recombinant *Af*Mnn9 and *Af*Van1 were shown to form a heterodimer and catalyze the synthesis of α-(1→6)-linked mannose polymers.

## RESULTS

### Mannosyltransferase activities of Mnn2 and Mnn5 *in vitro*

**Fig 1:**
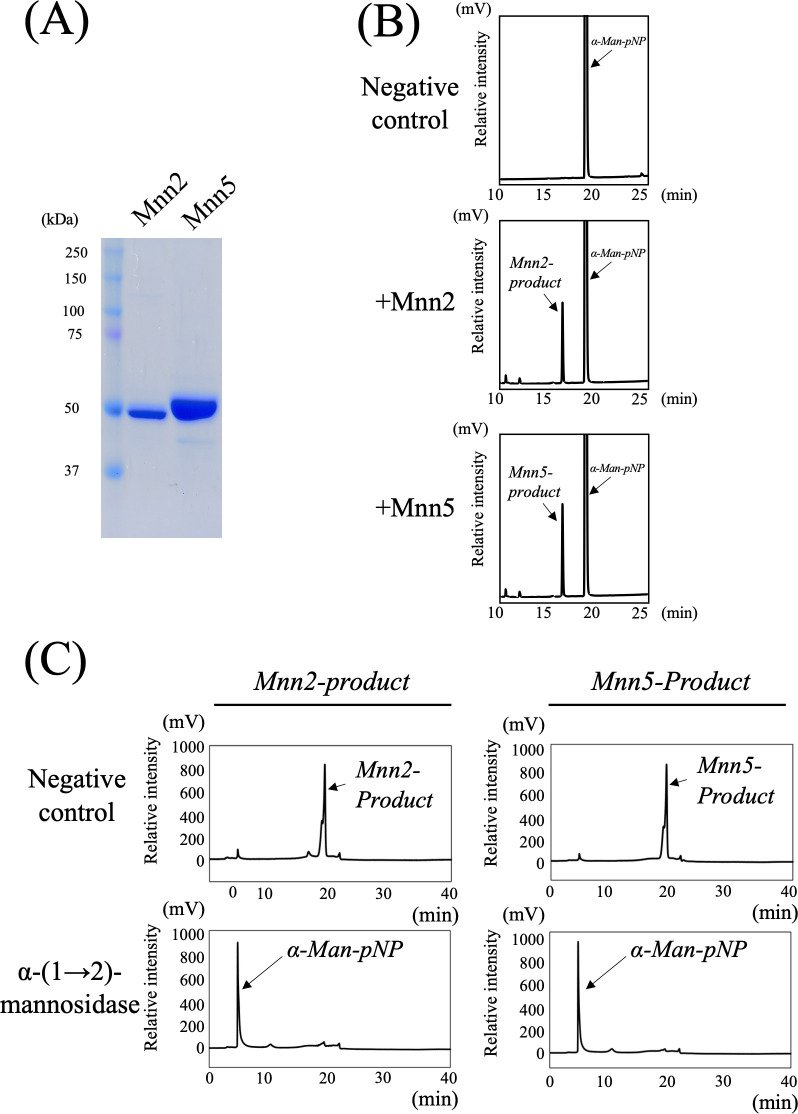
Mannosyltransferase activities of Mnn2 and Mnn5 *in vitro*. (**A**) SDS-PAGE analysis of purified recombinant Mnn2 and Mnn5. The purified recombinant proteins were separated by SDS-PAGE using a 5–20% gradient polyacrylamide gel. (**B**) Chromatograms of Mnn2 and Mnn5 mannosyltransferase activity assays using *p*-nitrophenyl α-D-mannopyranoside as the acceptor substrate. The 40 µL reaction mixture containing 50 mM HEPES-NaOH (pH 6.8), 100 mM NaCl, 30 mM KCl, 5% glycerol, 0.5 mM MnCl_2_, 1.5 mM α-Man-pNP (acceptor substrate), 10 mM GDP-Man (donor substrate), and 4.0 µg of purified Mnn2 and Mnn5 was incubated at 30°C for 16 h, respectively. Substrates and products were analyzed using reverse phase-HPLC. Chromatograms show typical results of the assay without enzyme (negative control, upper panel), with Mnn2 (middle panel), and with Mnn5 (lower panel). The assay without enzyme yielded only peaks derived from the α-Man-pNP at 16.8 min, whereas fractions with Mnn2 and Mnn5 displayed reaction products (termed Mnn2-product and Mnn5-product) at 19.5 min, respectively. (**C**) Structural analysis of Mnn2-product (at left) and Mnn5-product (at right) using α−1,2-mannosidase. These products were analyzed by normal-phase HPLC. Upper panels show chromatographs of the purified Mnn2-product or Mnn5-product. Purified Mnn2-product and Mnn5-product were reacted with α−1,2-mannosidase (lower panels). Both Mnn2-product and Mnn5-product could be reacted with α−1,2-mannosidase and digested to α-Man-pNP.

In our investigation of the mannosyltransferase activities of Mnn2 (AFUB_093840) and Mnn5 (AFUB_060800) from *A. fumigatus*, we produced individual N-terminal 6×His-tagged recombinant proteins using a bacterial expression system. Mnn2 and Mnn5 were expressed without the predicted transmembrane domains, spanning amino acid residues 1–30 and 1–29, respectively. Analysis of the purified recombinant proteins through sodium dodecyl sulfate-polyacrylamide gel electrophoresis (SDS-PAGE) revealed bands close to their predicted molecular weights of 53 and 52 kDa, respectively (Fig. 1A). Subsequently, we assessed mannosyltransferase activity at 30°C for 16 h using recombinant Mnn2 and Mnn5 (0.1 µg/µL each). The reaction involved *p*-nitrophenyl α-D-mannopyranoside (α-Man-pNP, 1.5 mM) as the acceptor substrate, guanosine diphosphate-α-D-mannose (GDP-Man, 10 mM) as the donor substrate, and 0.5 mM Mn^2+^ as the metal cofactor. Substrates and products were analyzed using reverse-phase-high-performance liquid chromatography (HPLC). Fractions with heat-inactivated Mnn2 and Mnn5 exhibited no new peaks (Fig. 1B, upper panels). Conversely, the fractions containing Mnn2 or Mnn5 revealed new products (referred to as Mnn2-product and Mnn5-product) at 19.5 min (Fig. 1B, middle and bottom panels). To elucidate the chemical structures of Mnn2-product and Mnn5-product, we isolated each peak and digested them using a substrate-specific mannosidase. These products were analyzed by normal-phase HPLC. Recombinant MsdS/MsdC, an α-(1→2)-specific mannosidase from *A. fumigatus*, was employed to convert Mnn2-product and Mnn5-product to α-Man-pNP (Fig. 1C). These findings provide compelling evidence supporting the role of Mnn2 and Mnn5 as α-(1→2)-mannosyltransferases.

The optimal temperature and pH for Mnn2 and Mnn5 reactions were determined. The optimal temperature was 40°C for Mnn2 and Mnn5, with activity decreasing sharply at >45°C (Fig. S1A). The highest enzyme activity was observed in 100  mM MES-NaOH buffer at pH 6.0, with Mnn2 and Mnn5 activity peaking at pH 5.5–6.5 (Fig. S1B). Next, we compared Mnn2 and Mnn5 activity using Mn^2+^, Ca^2+^, Co^2+^, Mg^2+^, Zn^2+^, and Cu^2+^ as cofactors, revealing that activity was highest when Mn^2+^ was the cofactor. Mnn2 exhibited moderate activity with Ca^2+^, Co^2+^, or Mg^2+^ (Fig. S1C). However, no activity was detected for Mnn2 or Mnn5 with the divalent cation chelator ethylenediaminetetraacetic acid (EDTA) (Fig. S1C).

### Colony morphology of *A. fumigatus* wild-type, Δ*mnn2*, Δ*mnn5*, and Δ*mnn2*Δ*mnn5* strains

**Fig 2:**
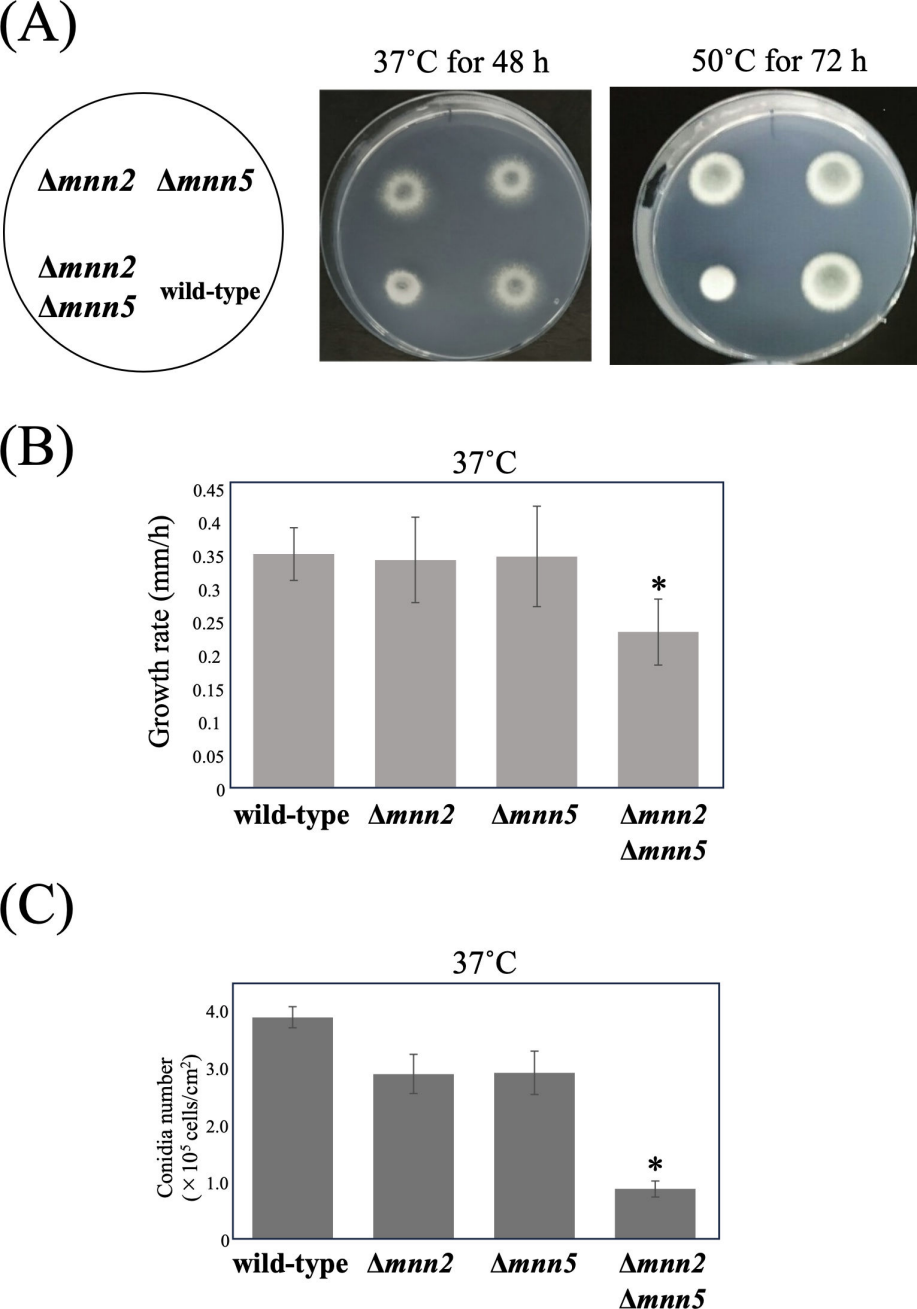
Colony morphology of *A. fumigatus* wild-type, Δ*mnn2*, Δ*mnn5*, and Δ*mnn2*Δ*mnn5.* (**A**) Colony morphology of wild-type (A1151), Δ*mnn2*, Δ*mnn5*, and Δ*mnn2* Δ*mnn5* on MM agar at 37 for 2 days and 50°C for 3 days, respectively. The agar medium was inoculated with 1.0 × 10^4^ conidiospores. (**B**) Colony growth rates of wild-type, Δ*mnn2*, Δ*mnn5*, and Δ*mnn2* Δ*mnn5* on MM agar at 37°C. (**C**) Conidia number per colony area of wild-type, Δ*mnn2*, Δ*mnn5*, and Δ*mnn2* Δ*mnn5* on MM agar at 37°C for 5 days. Asterisks indicate significant differences (*, *P* < 0.05; Welch’s t test) from the results for the wild-type strain.

To examine the physiological roles of Mnn2 and Mnn5 in *A. fumigatus*, we generated *A. fumigatus* strains with single and double mutations of *mnn2* and *mnn5* (Fig. S2A through S2C). Subsequently, we compared colony morphology at 37°C or 50°C on minimal media (MM). The Δ*mnn2* and Δ*mnn5* strains exhibited growth patterns similar to the wild-type A1151 strain (Fig. 2A), whereas ∆*mnn2*∆*mnn5* formed smaller colonies than wild-type (Fig. 2A). Additionally, colony growth rates were reduced (in mm/h, wild-type: 0.35 ± 0.04 vs. ∆*mnn2*∆*mnn5*: 0.23 ± 0.05 mm/h or 65.7% of wild-type; Fig. 2B). The Δ*mnn2*Δ*mnn5* strain also exhibited reduced conidia formation (Fig. 2C) and conidia number (conidia/mm^2^: wild-type: 3.89 × 10^5^ ± 1.85 × 10^4^, ∆*mnn2*∆*mnn5*: 8.82 × 10^4^ ± 1.40 × 10^4^ or 22.7% of wild-type; Fig. 2C); however, these two effects were rescued to almost wild-type levels by reintroduction of *mnn2* or *mnn5* into ∆*mnn2*∆*mnn5* (yielding complementary stain ∆*mnn2*∆*mnn5* +pPTR-II-Mnn2 or ∆*mnn2*∆*mnn5* +pPTR-II-Mnn5) (Fig. S3A through S3C). The colony growth rate and number of conidia were not significantly different between wild-type and Δ*mnn2* or wild-type and Δ*mnn5* strains (Fig. 2B and C). These findings indicate that Mnn2 and Mnn5 possess overlapping functions and play roles in the processes of hyphal elongation and conidia formation in *A. fumigatus*.

### Structural analysis of mannan from *A. fumigatus* wild-type, Δ*mnn2*, Δ*mnn5*, and Δ*mnn2*Δ*mnn5* strains

**Fig 3:**
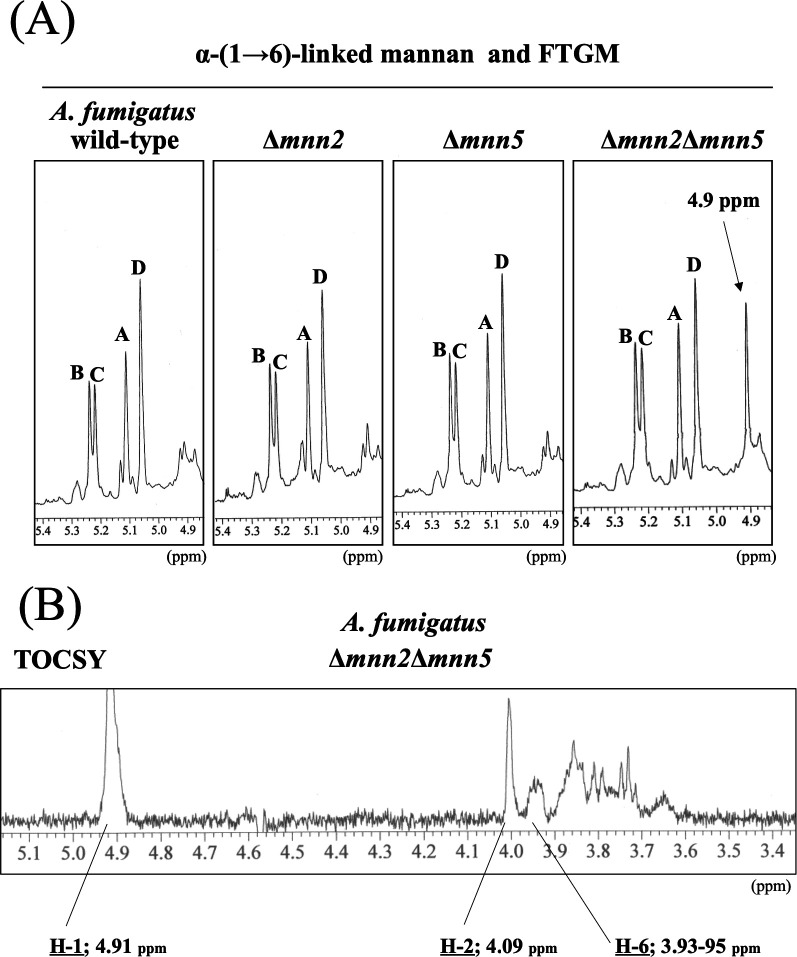
^1^H-NMR analysis of total mannan extracted from *A. fumigatus* wild-type, Δ*mnn2*, Δ*mnn5*, and Δ*mnn2*Δ*mnn5*. The total mannan fractions were extracted from wild-type (A1151), Δ*mnn2,* Δ*mnn5,* and Δ*mnn2*Δ*mnn5* strains. The signals A (at 5.104 ppm), B (5.233), C (5.216), and D (5.054) of the ^1^H-NMR spectra are derived from H-1 at the C-1 position of the underlined Man residues in the structures -(1→6)-α-Man-(1→2)-α-Man-(1→2)-α-Man-(1→2)-α-Man-(1→6)- (**A**), -(1→6)-α-Man-(1→2)-α-Man-(1→2)-α-Man-(1→2)-α-Man- (1→6)- (**B**), -(1→6)-α-Man-(1→2)-α-Man-(1→2)-α-Man-(1→2)-α-Man-(1→6)- (**C**), and -(1→6)-αMan-(1→2)-α-Man-(1→2)-α-Man-(1→2)-α-Man-(1→6)- (**D**). The signals at 4.9 ppm in the Δ*mnn2*Δ*mnn5* of the ^1^H-NMR spectra are from H-1 at the C-1 position of the underlined Man residue in the structures t-α-Man-(1→6)-α-Man and -(1→6)-α-Man-(1→6)-. The proton chemical shifts were referenced relative to internal acetone at δ 2.225 ppm.

To investigate the functions of Mnn2 and Mnn5 *in vivo*, we analyzed total mannan fraction from *A. fumigatus* wild-type, Δ*mnn2*, Δ*mnn5*, and Δ*mnn2*Δ*mnn5* strains by ^1^H-NMR. The total mannan were extracted from *A. fumigatus* mycelia by autoclaving at 121°C for 2 h with 100 mM citrate buffer (pH 7.0) (31). They were then treated with 0.1 M hydrochloric acid to remove the β-(1→5)-/β-(1→6)-galactofuran side chain. *O*-linked glycans, such as OMGM, were removed using the β-elimination method. The FTGM α-core-mannan structure was analyzed using ^1^H-NMR spectroscopy. The common four signals (A–D) of wild-type, Δ*mnn2*, Δ*mnn5*, and Δ*mnn2*Δ*mnn5* strains emerged, indicating the H-1 signal of the chemical shift of the α-(1→2)-/α-(1→6)-linked mannan of FTGM (Fig. 3A) (3). However, the 4.9 ppm signal, which indicates the H-1 signal of the chemical shift of the α-(1→6)-linked mannan, was specifically detected in the Δ*mnn2*Δ*mnn5* strain (Fig. 3A). The one-dimensional total correlation spectroscopy (TOCSY) spectrum of Δ*mnn2*Δ*mnn5,* recorded by the irradiation of the signal at 4.9 ppm corresponding to the branching α-(1→6)-mannosyl residue (Fig. 3B), was very similar to the previously reported spectrum of elongated α-(1→6)-linked mannan from *S. cerevisiae* ∆*Scmnn2* (32). These results suggest that the double disruption of *mnn2* and *mnn5* resulted in the α-(1→6)-linked mannan backbone becoming exposed and detectable in *A. fumigatus*.

### Expression of *Af*Mnn2 or *Af*Mnn5 in *S. cerevisiae* Δ*Scmnn2*Δ*Scmnn5* strain

**Fig 4:**
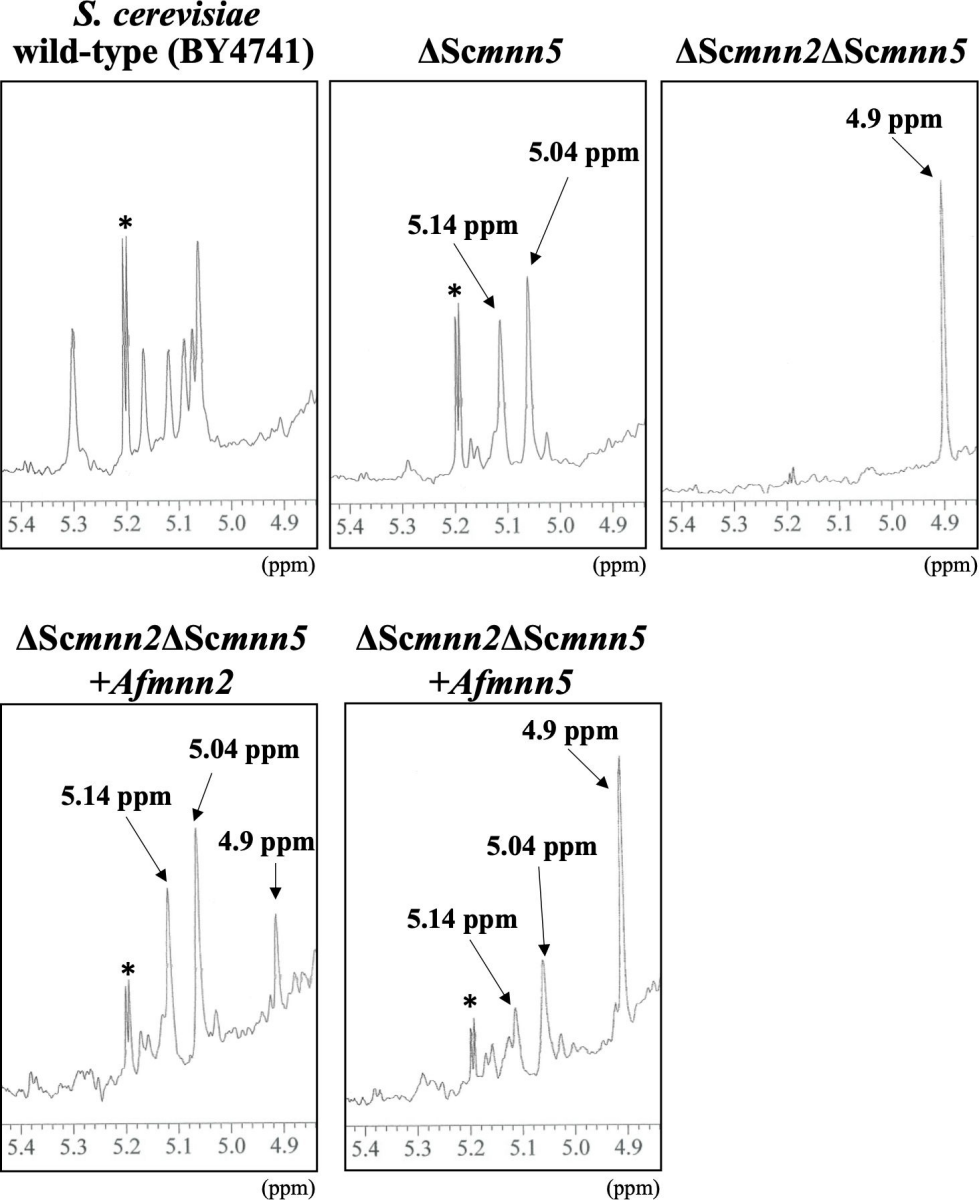
^1^H-NMR analysis of *N*-glycan outer chains from yeast wild-type (BY4741), ΔSc*mnn5*, ΔSc*mnn2*ΔSc*mnn5*, ΔSc*mnn2*ΔSc*mnn5 + mnn2*, and ΔSc*mnn2*ΔSc*mnn5 + mnn5* strains. The ^1^H-NMR spectra depict the *N*-glycan outer chains of various yeast strains. In the wild-type strain (BY4741), signals at 5.0 ~ 5.3 ppm correspond to H-1 at the C-1 position of Man residues in the α-(1→2)-Man and α-(1→3)-Man side chain of the outer chain. In Δ*Scmnn5*, signals at 5.04 and 5.14 ppm indicate H-1 at the C-1 position of Man in the structures t-α-Man-(1→2)-α-Man and Man-(1→2)-α-Man-(1→6)-α-Man. In Δ*Scmnn2*Δ*Scmnn5*, signals at 4.9 ppm are from H-1 at the C-1 position of the underlined Man residue in the structures t-α-Man-(1→6)-α-Man and -(1→6)-α-Man-(1→6)-. Asterisks denote unidentified NMR signals. Proton chemical shifts are referenced relative to internal acetone at δ 2.225 ppm.

To assess if *A. fumigatus mnn2* and *mnn5* can complement the Δ*Scmnn2*Δ*Scmnn5* strain, we developed heterologous *Af*Mnn2- or *Af*Mnn5-expressing strains in *S. cerevisiae* with a Δ*Scmnn2*Δ*Scmnn*5 genetic background. The outer chain structure of wild-type (BY4741), Δ*Scmnn2*, Δ*Scmnn5,* Δ*Scmnn2*Δ*Scmnn5*, Δ*Scmnn2*Δ*Scmnn5* + *Afmnn2*, and Δ*Scmnn2*Δ*Scmnn5* + *Afmnn5* strains was analyzed using ^1^H-NMR (Fig. 4). The chemical shift around 5.0–5.3 ppm observed in the wild-type indicates the presence of the α-(1→2)- and α-(1→3)-mannosyl side chain of the *N*-glycan outer chain (33). In Δ*Scmnn5*, chemical shifts appeared at 5.04 ppm and 5.14 ppm, indicating the presence of Man-α-(1→2)-Man and Man-α-(1→2)-Man-α-(1→6)-Man, respectively (33). Additionally, a chemical shift of 4.9 ppm was observed in Δ*Scmnn2*, indicating the presence of α-(1→6)-linked mannan (Fig. 4). Heterologous expression of *Af*Mnn2 and *Af*Mnn5 in Δ*Scmnn2*Δ*Scmnn5*, respectively, resulted in the appearance of chemical shifts at 5.04 and 5.14 ppm, although the chemical shift at 4.9 ppm was not completely eliminated (Fig. 4). The result indicates that *Af*Mnn2 and *Af*Mnn5 partially complement the function of the budding yeast *Sc*Mnn2p. These results suggest that the functions of *Af*Mnn2 and *Af*Mnn5 are the same as *Sc*Mnn2p and that they do not, or do very weakly, facilitate transfer of the second mannosyl residue.

### Structural analysis of α-(1→6)-linked mannan from *A. fumigatus* wild-type, Δ*mnn2*Δ*mnn5*, Δ*mnn2*Δ*mnn5*Δ*mnn9*, Δ*mnn2*Δ*mnn5*Δ*van1*, and Δ*mnn2*Δ*mnn5*Δ*anpA* strains

**Fig 5:**
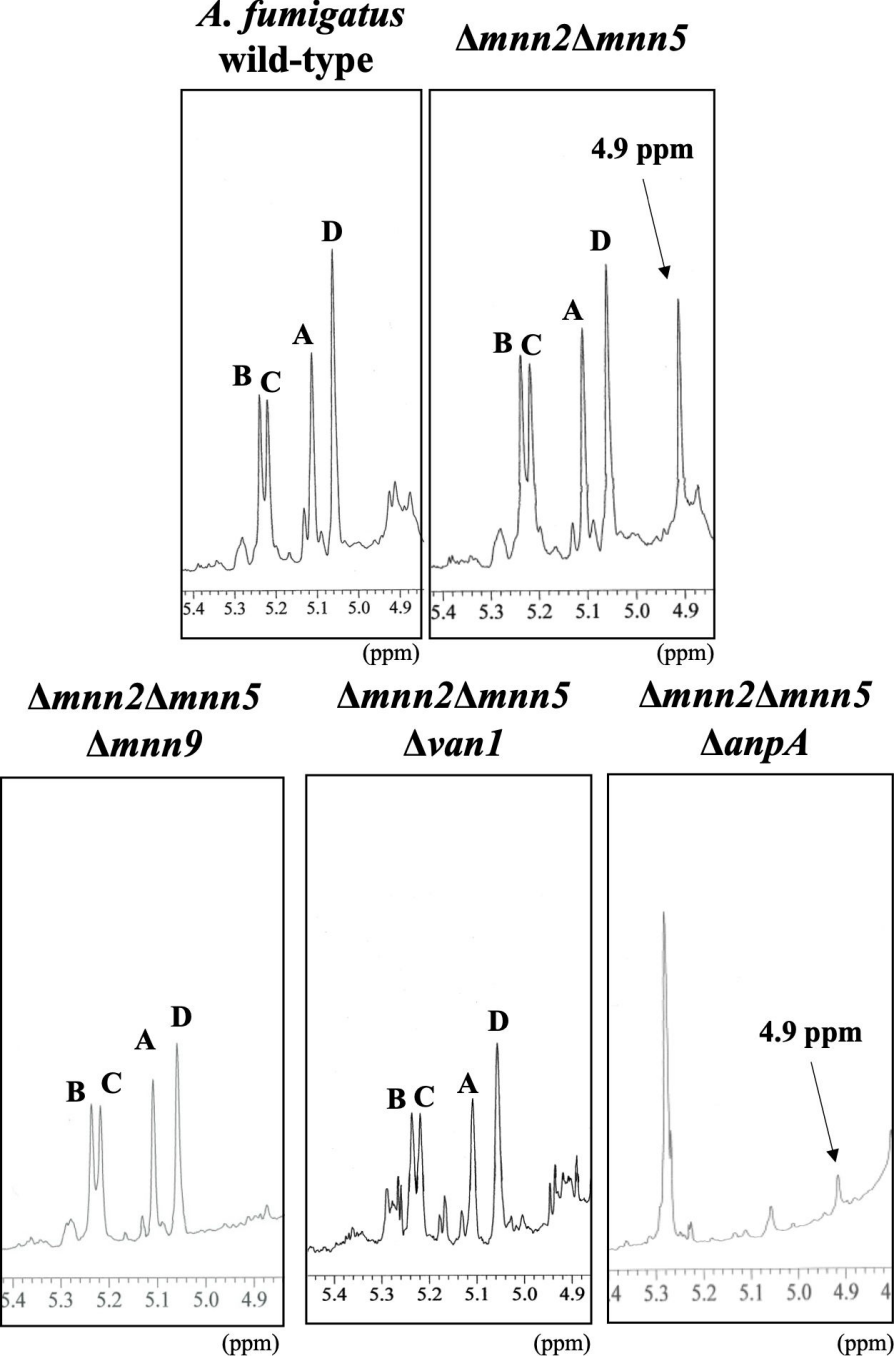
^1^H-NMR analysis of α-(1→6)-linked mannan from *A. fumigatus* wild-type, Δ*mnn2*Δ*mnn5*, Δ*mnn2*Δ*mnn5*Δ*mnn9*, Δ*mnn2*Δ*mnn5*Δ*van1*, and Δ*mnn2*Δ*mnn5*Δ*anpA*. The signals A (at 5.104 ppm), B (5.233), C (5.216), and D (5.054) of the ^1^H-NMR spectra are derived from H-1 at the C-1 position of the underlined Man residues in the structures -(1→6)-α-Man-(1→2)-α-Man-(1→2)- α-Man-(1→2)-α-Man-(1→6)- (**A**), -(1→6)-α-Man-(1→2)-α-Man-(1→2)-α-Man-(1→2)-α-Man- (1→6)- (**B**), -(1→6)-α-Man-(1→2)-α-Man-(1→2)-α-Man-(1→2)-α-Man-(1→6)- (**C**), and -(1→6)-αMan-(1→2)-α-Man-(1→2)-α-Man-(1→2)-α-Man-(1→6)- (**D**). The signals at 4.9 ppm in the Δ*mnn2*Δ*mnn5* of the ^1^H-NMR spectra are from H-1 at the C-1 position of the underlined Man residue in the structures t-α-Man-(1→6)-α-Man and -(1→6)-α-Man-(1→6)-. Asterisks indicate unidentified NMR signals. Proton chemical shifts are referenced relative to internal acetone at δ 2.225 ppm.

To identify the glycosyltransferases involved in the biosynthesis of *A. fumigatus*, we extracted the total mannan from wild-type, Δ*mnn2*Δ*mnn5*, Δ*mnn2*Δ*mnn5*Δ*mnn9*, Δ*mnn2*Δ*mnn5*Δ*van1,* and Δ*mnn2*Δ*mnn5*Δ*anpA* strains and analyzed their structures (Fig. 5). The disruption of *mnn9* or *van1* in the Δ*mnn2*Δ*mnn5* strain resulted in the disappearance of the 4.9 ppm chemical shift observed in Δ*mnn2*Δ*mnn5* (Fig. 5). This suggests that Mnn9 or Van1 are essential for the biosynthesis of α-(1→6)-linked mannan in *A. fumigatus*. In the Δ*mnn2*Δ*mnn5*Δ*anpA* strain, the signal of FTGM was absent, but the signal of α-(1→6)-linked mannan was unaffected. This indicates that AnpA is involved only in the biosynthesis of FTGM, but not α-(1→6)-linked mannan in *A. fumigatus* (Fig. 5).

### Is α-(1→6)-linked mannan bound to the outer chain of *N*-glycan?

Next, we evaluated the involvement of Mnn2, Mnn5, Mnn9, and Van1 in *N*-glycan biosynthesis in *A. fumigatus* using a *N*-glycosylated protein, SucA. The SucA was detected by immunoblotting to estimate the length of *N*-glycan. If these mannosyltransferases contribute to *N*-glycan elongation, a lower apparent molecular weight should be observed in these strains than in the A1151 strain, as SucA has nine potential *N*-glycosylation sites. SucA was detected as two major bands, including one high- and one low-molecular-weight band. Both main bands had increased mobility following endoglycosidase (Endo) H treatment, indicating that these SucA proteins were *N*-glycosylated (Fig. S4, lanes 1 and 5). The apparent molecular weight of both types of SucA expressed in ∆*mnn2*∆*mnn5*, ∆*mnn9,* and ∆*van1* strains was comparable with SucA expressed in A1151 (Fig. S4, lanes 1–4). This suggests that the α-(1→6)-linked mannan is derived from a glycan different from the *N*-glycan outer chain or a short *N*-glycan outer chain structure.

### Mannosyltransferase activities of Mnn9 and/or Van1 *in vitro*

**Fig 6:**
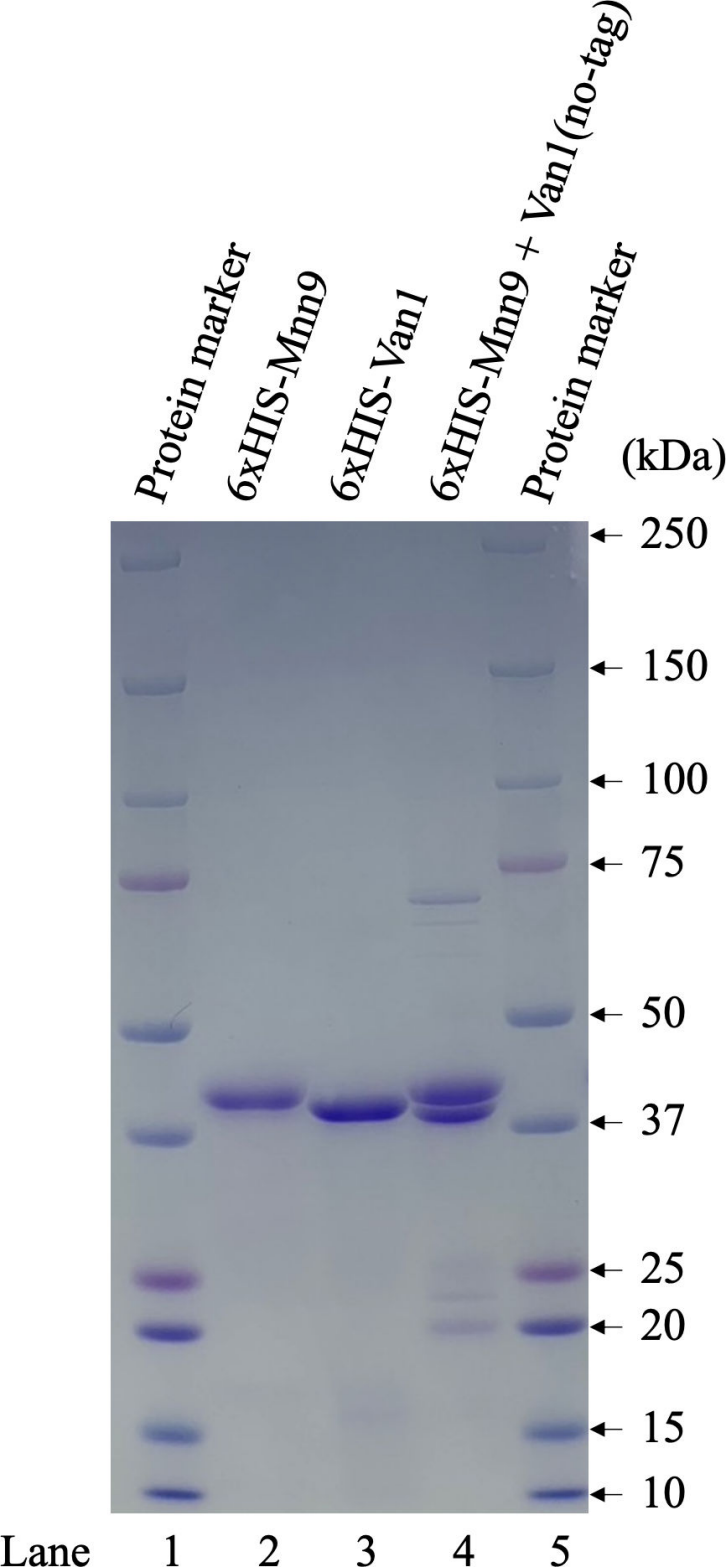
Recombinant proteins of Mnn9, Van1, and Mnn9-Van1 complex. SDS-PAGE analysis of purified recombinant 6×His-tagged Mnn9, 6×His-tagged Van1, and 6×His-tagged Mnn9 + non-tagged Van1. Lanes 2 and 3 show 3 µg of purified proteins (lane 2: 6×His-tagged Mnn9 and lane 3: 6×His-tagged Van1). Lane 4 shows 6 µg of purified proteins (lane 4: 6×His-tagged Mnn9 + non-tagged Van1). Lanes 1 and 5 display 5 µL of Precision Plus Protein Dual Color Standard (Bio-Rad).

**Fig 7:**
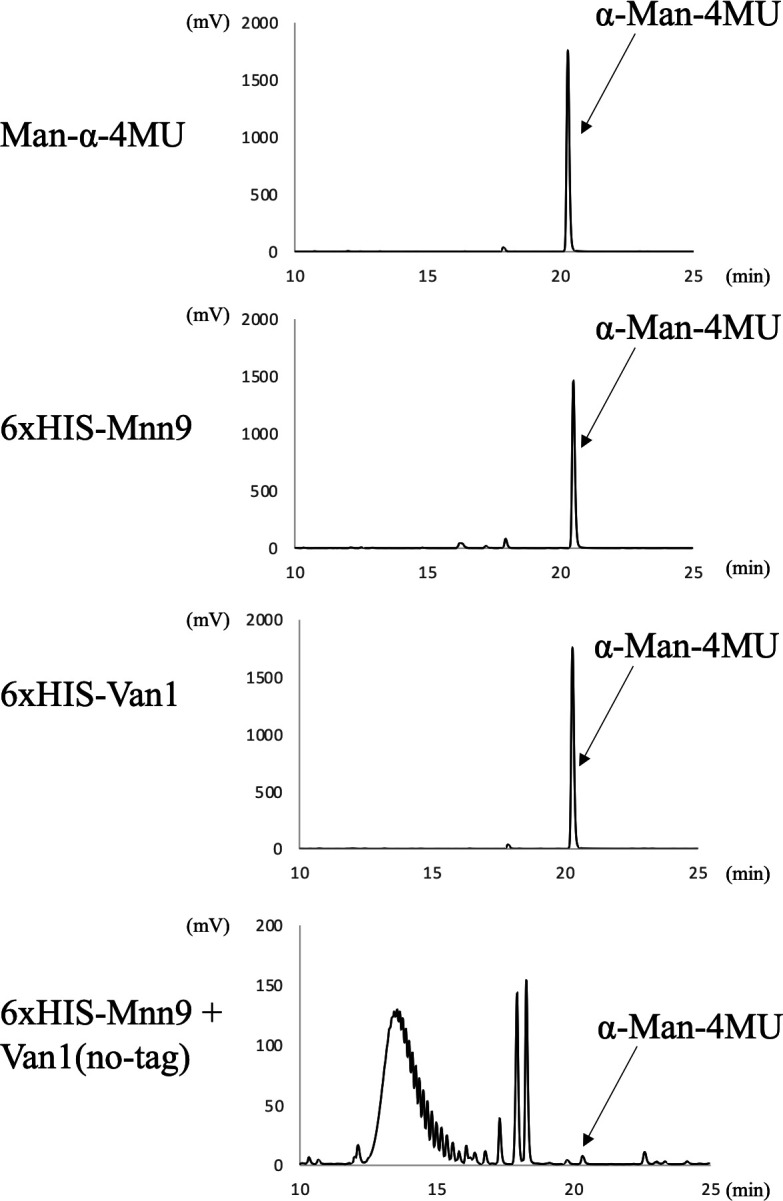
Mannosyltransferase activities of Mnn9 and/or Van1 *in vitro*. Chromatograms of Mnn9 and/or Van1 mannosyltransferase activity assays using 4-methylumbelliferyl α-D-mannopyranoside (α-Man-4MU) as the acceptor substrate. The assays were conducted in a reaction mixture (40 µL) containing 50 mM HEPES-NaOH (pH 6.8), 100 mM NaCl, 30 mM KCl, 5% glycerol, 0.5 mM MnCl_2_, 1.5 mM α-Man-4MU (acceptor substrate), 10 mM GDP-Man (donor substrate), and 4 µg of purified enzymes at 30°C for 16 h. Chromatograms depict results of the assay without enzyme (negative control) and with 6×His-tagged Mnn9, 6×His-tagged Van1, and 6×His-tagged Mnn9 + non-tagged Van1, respectively. Without enzyme and Mnn9 alone, Van1 alone assays show peaks derived from α-Man-4MU at 21.0 min and fractions with 6×His-tagged Mnn9 + non-tagged Van1 display reaction products.

**Fig 8:**
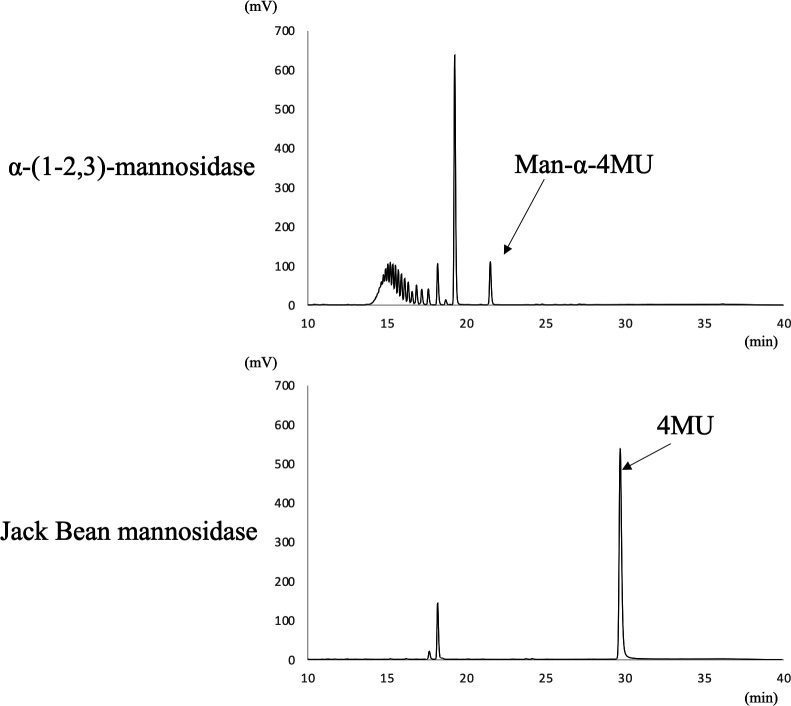
Structural analysis of Mnn9-Van1 complex. Structural analysis of 6×His-tagged Mnn9 + non-tagged Van1 products using α-(1→2,3)-mannosidase and Jack Bean mannosidase. Purified 6×His-tagged Mnn9 + non-tagged Van1 product was reacted with α-(1→2,3)-mannosidase (upper panel) and Jack Bean mannosidase (lower panel). Both 6×His-tagged Mnn9 + non-tagged Van1 product could be reacted with Jack Bean mannosidase and digested to 4MU.

To investigate whether Mnn9 and Van1 induce α-(1→6)-linked mannan *in vitro*, we generated individual N-terminal 6×His-tagged recombinant proteins using a bacterial expression system. Mnn9 and Van1 were expressed without the presumed transmembrane domains, covering amino acid residues 1–35 and 1–69, respectively. Although Mnn9 yielded solubilized protein successfully (Fig. 6, lane 2), unfortunately, Van1 proved to be insoluble (data not shown). In an attempt to address this, we also removed the C-terminal region of Van1 (amino acid residues 387–499), which is not homologous to AfMnn9. This modification led to the successful purification of solubilized Van1 protein (Fig. 6, lane 3). Mnn9 and Van1 did not exhibit mannosyltransferase activity when assayed individually (Fig. 7). Subsequently, we co-expressed 6×His-tagged Mnn9 and untagged Van1 (Fig. 6, lane 4). Purification with Ni-agarose yielded Van1 along with Mnn9, even without the 6×HIS tag on Van1 (Fig. 6, lane 4). This indicated the formation of a protein complex between Mnn9 and Van1. Interestingly, when assayed for mannosyltransferase activity *in vitro*, multiple peaks, potentially representing mannosyl polymers, were detected under the Mnn9 and Van1 co-expression conditions (Fig. 7). This peak was susceptible to digestion by jack bean mannosidase (α-(1→2,3,6)-mannosidase) but not by α-(1→2,3)-mannosidase, conclusively confirming it as an α-(1→6)-mannose polymer (Fig. 8). These results suggest that Mnn9 and Van1 exclusively function as mannan polymerases *in vitro* when they form protein complexes.

## DISCUSSION

In this investigation, we delved into the functional roles of Mnn2 and Mnn5, contributors to hyphal growth and conidial formation through the biosynthesis of mannan structures in *A. fumigatus*.

**Fig 9:**
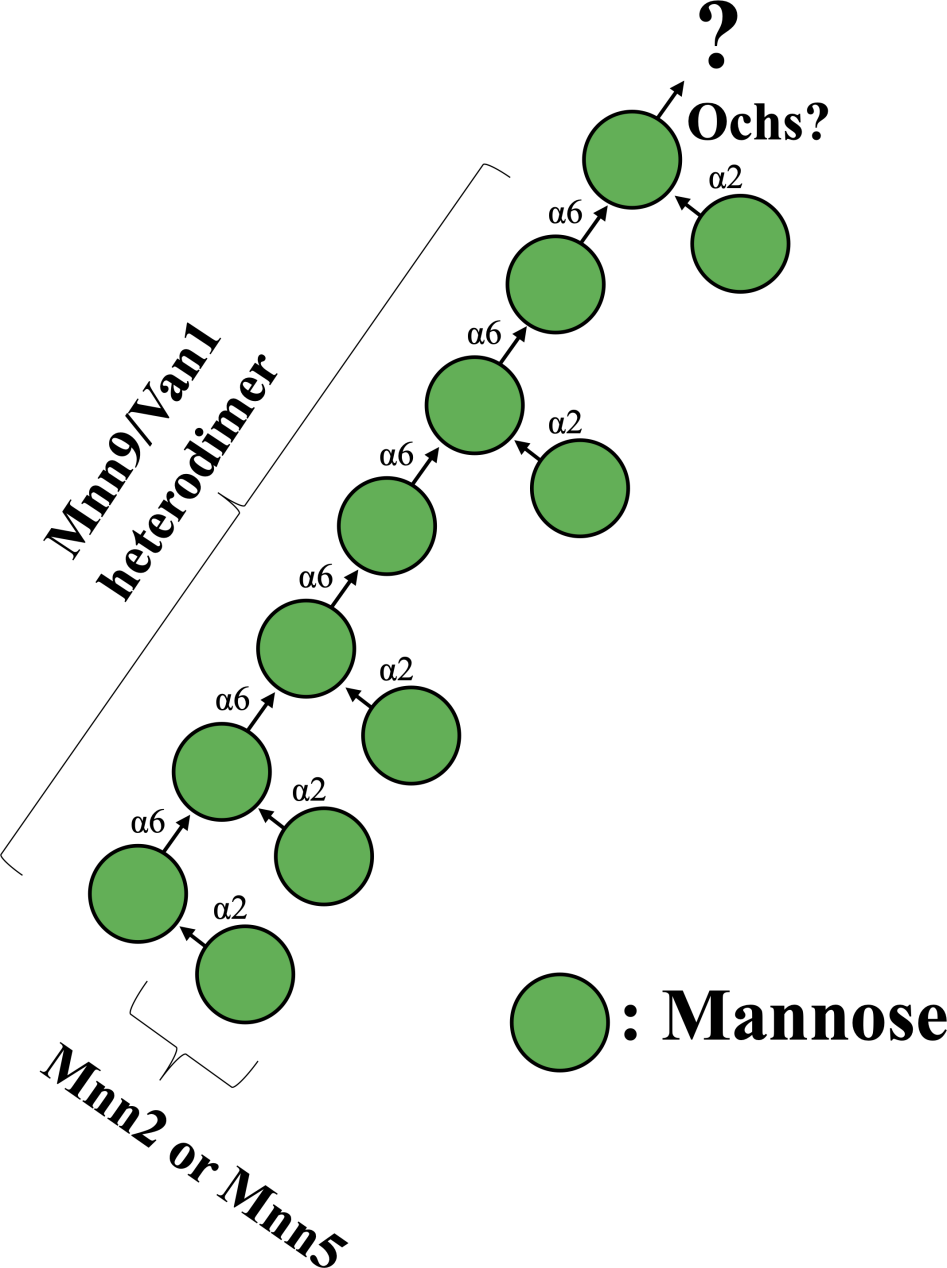
Summary model of α-(1→6)-linked mannan biosynthesis in *A. fumigatus*. Ochs represent the initial specific α-(1→6)-mannosyltransferase. Mnn9-Van1 complex represents α-(1→6)-mannan polymerase. Mnn2 and Mnn5 represent α-(1→2)-mannosyltransferase.

The ^1^H-NMR analysis of the total mannan fraction unveiled the presence of α-(1→6)-linked mannan structures in *A. fumigatus* mycelium. The distinctive chemical shift originating from the α-(1→6)-linked mannan becomes discernible when the side chain is absent (22, 32–34). Both *Af*Mnn2 and *Af*Mnn5 emerge as pivotal catalysts in side-chain biosynthesis, indicating their collaborative role in transferring the initial α-(1→2)-mannosyl residues to the α-(1→6)-linked mannan backbone (Fig. 9). In *S. cerevisiae*, *Sc*Mnn5p is recognized for transferring the second α-(1→2)-mannosyl residue of the side chain (18). However, upon heterologous expression of *Af*Mnn2 or *Af*Mnn5, the mannan structure of Δ*Scmnn2*Δ*Scmnn5* was restored to that of Δ*Scmnn5* (Fig. 4), indicating that *Af*Mnn2 and *Af*Mnn5 share the same function as *Sc*Mnn2p; however, they do not have the enzymatic function of *Sc*Mnn5p. This suggests that the chain length of the α-(1→2)-mannosyl side chain of α-(1→6)-linked mannan may be limited to one in *A. fumigatus* (Fig. 9). The detailed structural analysis of this glycan in *A. fumigatus* could clarify this conclusion. We uncovered the involvement of Mnn9 and Van1, members of the GT62 family, in mycelial α-(1→6)-linked mannan biosynthesis (Fig. 5). The recombinant heterodimer of Mnn9 and Van1 was found to function as a mannan polymerase *in vitro* (Fig. 6 to 8). Contrary to prior investigations documenting Mnn9’s manifestation of mannosyltransferase activity in isolation (28), our results suggest that neither Mnn9 nor Van1 has mannosyltransferase activity independently *in vitro* (Fig. 7). The present study used lower enzyme concentrations, lower substrate and substrate manganese concentrations, and different reaction conditions (e.g., omission of DTT) than Henry et al, which could account for the differences in recorded enzyme activity (28). However, the homologous GT62-family protein in *A. fumigatus*, AnpA, exhibits strong mannosyltransferase activity alone under the reaction conditions used in our previous study (35). Henry et al. may have observed very weak activity (28). Furthermore, our results align with the obliteration of the α-(1→6)-linked mannan-derived chemical shift following the singular disruption of either *mnn9* or *van1* (Fig. 5). These findings underscore the essential collaborative role of both *mnn9* and *van1* in the biosynthesis of the α-(1→6)-linked mannan in *A. fumigatus*. In the context of *A. fumigatus*, the *mnn2* and *mnn5* double-disruption strain displayed a reduced mycelial elongation rate and diminished ability to form conidiophores (Fig. 2). Notably, reports indicate that Δ*mnn9* and Δ*van1* do not exhibit the Δ*mnn2*Δ*mnn5*-like phenotype (28, 35). This discrepancy suggests that Mnn2 and Mnn5 may also be involved in the biosynthesis of mannans beyond the α-(1→6)-linked mannan side chains. Mnn2 and Mnn5 are potentially involved in the biosynthesis of mannosyl residues in *A. fumigatus* glycolipids. However, *A. fumigatus* strains deficient in mannosyltransferase or *N*-acetylglucosaminyltransferase to inositol phosphorylceramide exhibit normal mycelial growth (36–38), suggesting that Mnn2 and Mnn5 are unlikely to be involved in glycolipid biosynthesis. The intricate landscape of mannan biosynthesis in *A. fumigatus* remains complex and warrants further elucidation in future studies.

Mnn9 and Van1 in *A. fumigatus* have undergone extensive scrutiny, with recent findings linking them to the biosynthesis of the conidia-specific G3Man structure (30). Our study contributes a novel revelation by showcasing, for the first time, the presence of α-(1→6)-linked mannan in both mycelia and conidia. Intriguingly, Mnn9 and Van1 play a role in the biosynthesis of α-(1→6)-linked mannan in mycelial and conidial forms (30). However, the structural disparities in the side chains between conidial and mycelial α-(1→6)-linked mannans suggest that, in comparison to the complex G3Man, the mycelial α-(1→6)-linked mannan exhibits a simpler structure (Fig. 9). This structural distinction is likely attributed to the necessity for a complex structure, such as G3Man, in conidia due to the high percentage of β-glucan and the absence of galactosaminogalactan (GAG) (30). Conversely, the presence of α-glucan and GAG in the mycelium may render a simpler mannan structure sufficient. The presence of mannans with different side-chain structures in the cell walls of conidia and mycelia remains unparalleled among known polysaccharides. Therefore, investigating the specific functional roles of these mannans in conidia and mycelia presents an intellectually compelling avenue for research. The physiological significance of these mannan structures warrants further elucidation.

To determine whether the α-(1→6)-linked mannan structure of *A. fumigatus* is part of the *N*-glycan, we investigated the changes in the apparent molecular weight of the invertase SucA on SDS-PAGE (Fig. S4). Loss of outer chains of *N*-glycans in budding and fission yeasts dramatically reduced the apparent molecular weight of *N*-glycosylated proteins on SDS-PAGE, such as invertase and acid phosphatase (12, 39, 40). However, in *A. fumigatus*, the apparent molecular weight change of SucA upon Endo H treatment was smaller than that in yeast (Fig. S4, lanes 1 and 5), consistent with those reported by other groups (28, 29). These results suggest that the *N*-glycans of *A. fumigatus* do not possess the long outer chain structure present in the yeast. Moreover, we observed no remarkable change in the mobility of SucA in the Δ*mnn2*Δ*mnn5*, ∆*mnn9,* and ∆*van1* strains compared with that in A1151 (Fig. S4, lanes 1–4). This may be because the *N*-glycosylated proteins of *A. fumigatus* do not have an *N*-glycan outer chain or as the *N*-glycans of *A. fumigatus* are not as long as those of yeast. In this paper, no conclusions could be drawn as to what the α-(1→6)-linked mannan structure is bound to. Du et al. proposed that α-mannan elongation occurs not via *N*-glycan but through *O*-linked mannose in cell wall mannoproteins (29). Unlike yeasts, which heavily rely on outer chain structures for cell wall integrity, the presence of other mannan structures, such as FTGM, in *A. fumigatus* may diminish the importance of outer chain structures. Conversely, certain species, such as *N. crassa,* emphasize the significance of outer chain structure even in filamentous fungi, showcasing the intriguing diversity of mannan structures, including the outer chain, within the phylum Ascomycota (25). In filamentous fungi, different mannans are present in the cell wall. Because the structures of the mannans are similar, distinguishing between and analyzing them is difficult. Further studies are needed to clarify their differences.

In summary, our investigations into Mnn2 and Mnn5 have unveiled the presence of an α-(1→6)-linked mannan structure in the mycelia of *A. fumigatus*. These findings mark a substantial contribution to our comprehension of the structure and biosynthesis of filamentous fungal cell wall, with potential implications for the development of antifungal and agrochemical agents.

## MATERIALS AND METHODS

### Strains and medium

The *A. fumigatus* strains utilized in this investigation are detailed in Table S1 (Table S1), with *A. fumigatus* A1160 and A1151 strains serving as the parental and control strains, respectively.

Cultures were grown on *Aspergillus* minimal medium (1% [wt/vol] glucose, 0.6% [wt/vol] NaNO_3_, 0.052% [wt/vol] KCl, 0.052% [wt/vol] MgSO_4_·7H_2_O, and 0.152% [wt/vol] KH_2_PO_4_, supplemented with Hutner’s trace elements [pH 6.5]). For the A1160 strain cultivation, 1.22 g/L and 1.21 g/L uracil and uridine were incorporated into the MM.

The *S. cerevisiae* strains employed in this study are enumerated in Table S1. BY4741 and Δ*mnn5* strains were procured from the Yeast Knockout (YKO) Collection (Horizon Discovery Ltd., UK). Growth for these strains occurred on yeast extract-peptone-dextrose (YPD) medium or synthetic complete (SC) medium.

### Construction of *mnn2* and *mnn5* gene disruption strains

*A. fumigatus mnn2* or *mnn5* mutants were made by replacing *mnn2* with *mnn2*Δ::*AnpyrG* or *mnn5*Δ::*AnpyrG* cassettes. A gene replacement cassette, consisting of the homology arm at the 5′ end of the *mnn2* and *mnn5* genes and the homology arm at the 3′ end of the *mnn2* and *mnn5* genes, was generated by recombinant polymerase chain reaction (PCR). The *A. fumigatus* A1151 genomic DNA served as the template, and the primer pairs xxxx-1/xxxx-2 and xxxx-3/xxxx-4 were used for amplification (where “xxxx” indicates *mnn2* or *mnn5*) (Table S2). Simultaneously, the *AnpyrG* marker was amplified by recombinant PCR using pHSG396-AnpyrG (35) as the template and the primer pair pHSG396-F/pHSG396-R. The resulting DNA fragment, amplified with primers xxxx-1 and xxxx-4, was employed for the transformation of *A. fumigatus* A1160, yielding Δ*mnn2* and Δ*mnn5* strains. MM agar plates lacking uracil and uridine were utilized for the selection of transformants. The successful introduction of *AnpyrG* into each gene locus was confirmed through PCR, using the primer pairs mnn2-F/pyrG-R and pyrG-F/mnn2-R, as well as mnn5-F/pyrG-R and pyrG-F/mnn5-R, respectively (Fig. S2A and B).

### Construction of *∆mnn2∆mnn5* strains

*A. fumigatus ∆mnn2*∆*mnn5* mutants were made by replacing *mnn5* with *mnn5*Δ::*ptrA* or *mnn5*Δ::*hph* cassettes in *A. fumigatus ∆mnn2*. A gene replacement cassette, including the homology arm at the 5′ end of the *mnn5* gene and the homology arm at the 3′ end of the *mnn5* gene, was generated by recombinant PCR using *A. fumigatus* A1151 genomic DNA as the template and the primer pairs mnn5-1/mnn5-2 and mnn5-3/mnn5-4, respectively (Table S2). The *ptrA* and *hph* markers were amplified by recombinant PCR using pHSG396-ptrA and pHSG396-hph (10) as templates and the primer pair pHSG396-F/pHSG396-R. The resulting DNA fragments, amplified with primers mnn5-1 and mnn5-4, were used to transform *A. fumigatus* Δ*mnn2*, generating Δ*mnn2*Δ*mnn5* (*ptrA*) and Δ*mnn2*Δ*mnn5* (*hph*), respectively. MM agar plates supplemented with 0.1 µL/mL pyrithiamine or 200 µL/mL hygromycin B were employed for the selection of transformants. The introduction of *ptrA* or *hph* into each gene locus was confirmed by PCR using the primer pairs mnn5-F/ptrA-R and ptrA-F/mnn5-R, mnn5-F/hph-R, and hph-F/mnn5-R, respectively (Fig. S2C and D).

### Construction of the complementary strains ∆*mnn2*Δ*mnn5*+pPTR-II-Mnn2 or ∆*mnn2*Δ*mnn5*+pPTR-II-Mnn5

The *A. fumigatus* ∆*mnn2*∆*mnn5*(*hph*) strain was complemented with *Afmnn2* or *Afmnn5*. The *mnn2* or *mnn5* genes, including 1.0 kbp upstream and 0.5 kbp downstream of *mnn2* or *mnn5,* were amplified by PCR using *A. fumigatus* A1160 genomic DNA as the template and pPTR-II-mnn2-F/pPTR-II-mnn2-R or pPTR-II-mnn5-F/pPTR-II-mnn5-R as primer pairs. The amplified fragment was inserted into the SmaI site of pPTR-II using the In-Fusion HD cloning kit (Takara, Japan) to yield pPTR-II-Mnn2 or pPTR-II-Mnn5. The constructed plasmids were transformed into the ∆*mnn2*∆*mnn5*(*hph*) strain, yielding ∆*mnn2*Δ*mnn5* +pPTR-II-Mnn2 or ∆*mnn2*Δ*mnn5* +pPTR-II-Mnn5.

### Construction of Δ*mnn2*Δ*mnn5*Δ*mnn9*, Δ*mnn2*Δ*mnn5*Δ*van1*, and Δ*mnn2*Δ*mnn5*Δ*anpA* strains

The *mnn9*, *van1*, or *anpA* were disrupted in *A. fumigatus* Δ*mnn2*Δ*mnn5* (*ptrA*) by *hph* insertion. A gene replacement cassette, including the homology arm at the 5′ end of the *mnn9*, *van1,* or *anpA* genes and the homology arm at the 3′ end of the *mnn9*, *van1*, or *anpA* genes, was generated by recombinant PCR using *A. fumigatus* A1151 genomic DNA as the template and the primer pairs xxxx-1/xxxx-2 and xxxx-3/xxxx-4, respectively (where “xxxx” indicates *mnn9*, *van1*, or *anpA*) (Table S2). The *hph* marker was amplified by recombinant PCR using pHSG396-hph (10) as the template and the primer pairs pHSG396-F/pHSG396-R. The resulting DNA fragment, amplified with primers xxxx-1 and xxxx-4, was used to transform *A. fumigatus* A1160, generating Δ*mnn2*Δ*mnn5*Δ*mnn9*, Δ*mnn2*Δ*mnn5*Δ*van1*, or Δ*mnn2*Δ*mnn5*Δ*anpA* strains, respectively. MM agar plates supplemented with 200 µL/mL hygromycin B were employed for the selection of transformants. The introduction of *hph* into each gene locus was confirmed by PCR using the primer pair xxxx-F/hph-R and hph-F/xxxx-R (Fig. S2E thorugh G).

### Construction of Δ*Scmnn2*Δ*Scmnn5* in *S. cerevisiae*

The *Scmnn2* gene was disrupted in *S. cerevisiae* Δ*Scmnn5* by *HIS3* insertion. A gene replacement cassette, consisting of 50 bp of the homology arm at the 5′ end of the *mnn2* gene, *HIS3*, and 50 bp of the homology arm at the 3′ end of the *mnn2* gene, was amplified by PCR using pRS313 plasmid DNA as the template and the primer pairs ScMNN2-HIS3-F/ScMNN2-HIS3-R (Table S2). SC agar plates without uracil and uridine were employed for the selection of transformants. The introduction of *HIS3* into each gene locus was confirmed by PCR using the primer pairs ScMNN2-F/HIS-R and HIS-F/ScMNN2-R.

### Construction of YEp352-AfMnn2 and YEp352-AfMnn5 plasmids

The expression plasmid YEp352-GAP-II was employed as the vector for expressing AfMnn2 and AfMnn5 in *S. cerevisiae* strains. It is worth noting that for AfMnn2, the transmembrane region of *Sc*Mnn2p (amino acids 1 to 26) was fused with AfMnn2 (amino acids 31–493), and for AfMnn5, the transmembrane region of *Sc*Mnn5p (amino acids 1 to 35) was fused with AfMnn5 (amino acids 30–492).

For the construction of YEp352-AfMnn2, the DNA fragment encompassing the transmembrane region of *Sc*Mnn2p was PCR-amplified using genomic DNA extracted from *S. cerevisiae* BY4741 strain as the template, with the primer pair ScMnn2p-TM (1–26)-F/ScMnn2p-TM (1–26)-R. Subsequently, the AfMnn2 fragment was amplified using genomic DNA from the *A. fumigatus* A1151 strain as the template, with the primer pair YEp352-GAPII-Mnn2-F/YEp352-GAPII-Mnn2-R. Additionally, the 3×FLAG fragment was amplified using the pDC1-3× FLAG plasmid (31) as a template and the primer pair 3×FLAG-F/3×FLAG-R. These three amplified fragments were then seamlessly integrated into the *Sac* I site of YEP352-GAPII (41) using the In-Fusion HD cloning kit (Takara). Similarly, for the generation of YEp352-AfMnn5, the DNA fragment covering the transmembrane region of *Sc*Mnn5p was PCR-amplified using genomic DNA from *S. cerevisiae* BY4741 strain as the template, with the primer pair ScMnn5p-TM (1–35)-F/ScMnn5p-TM (1–35)-R. Next, the AfMnn5 fragment was amplified using genomic DNA from the *A. fumigatus* A1151 strain as the template, with the primer pair YEp352-GAPII-Mnn5-F/YEp352-GAPII-Mnn5-R. The two amplified fragments, together with the 3×FLAG fragment, were cloned into the *SacI* site of YEp352-GAP-II using the In-Fusion HD cloning kit. The transformation of budding yeast was performed using the lithium acetate method.

### Construction of pHSG396-ptrA plasmid

The pyrithiamine resistance gene (*ptrA*) was amplified by PCR, utilizing the plasmid pPTR I (TAKARA, Japan) as a template and the primer pairs pHSG396-ptrA-IF-F and pHSG396-ptrA-IF-R. Subsequently, the amplified fragment was incorporated into the *Bam* HI site of pHSG396 using the In-Fusion HD cloning kit (TAKARA, Japan), resulting in the generation of pHSG396-ptrA.

### Construction of pET15-SmaI plasmid

The adapted plasmid fragments were amplified via PCR, employing pET15-KAI (42) as a template with the primer pairs pET15-SmaI-F and pET15-SmaI-R. Subsequently, the amplified DNA fragments were self-ligated using the In-Fusion HD cloning kit (TAKARA, Japan) to generate pET15-SmaI.

### Construction of pET15-Mnn2, pET15-Mnn5, pET15-Mnn9, and pET15-Van1 plasmids

As the *mnn2* and *mnn5* genes lack introns, PCR amplification was conducted using genomic DNA from the *A. fumigatus* A1151 strain as a template, with primer pairs pET15-Mnn2-F, pET15-Mnn2-R, pET15-Mnn5-F, and pET15-Mnn5-R, respectively. For the *mnn9* genes, PCR amplification utilized total cDNA prepared from *A. fumigatus* A1151 with the primer pair pET15-Mnn9-F and pET15-Mnn9-R. The resulting DNA fragments were inserted into the *Nde* I and *Not* I sites of pET15-KAI (42) using the In-Fusion HD cloning kit, yielding pET15-Mnn2, pET15-Mnn5, and pET15-Mnn9. The *Escherichia coli* codon-optimized *van1* gene fragments from *A. fumigatus* A1163 were synthesized by gBlocks (Integrated DNA Technologies, USA) and cloned into the *Sma* I site of pET15-SmaI using the In-Fusion HD cloning kit, resulting in pET15-Van1. Construction of the plasmid for expressing the C-terminal truncated form (∆387–499) of *van1* involved PCR amplification of the *van1* (∆387–499) gene using pET15-Van1 as a template with the primer pair pET15-SmaI-Van1(∆387–499)-F and pET15-SmaI-Van1(∆387–499)-R. The amplified DNA fragments were then cloned into the *Sma* I site of pET15-SmaI using the In-Fusion HD cloning kit, yielding pET15-Van1(∆387–499). Subsequently, the plasmids were transformed into SHuffle T7 Express (New England Biolabs, USA) containing the plasmid pRARE extracted from Rosetta-gami B(DE3) (MilliporeSigma, Burlington, MA).

### Construction of pET15-AfMnn9-AfVan1 (no-tag) plasmid

The *mnn9* or *van1* genes were amplified by PCR using pET15-Mnn9 and pET15-Van1(∆387–499) as templates, with primer pairs pET15-SmaI-Mnn9-Van1(∆387–499)(no-tag)-F1, pET15-SmaI-Mnn9-Van1(∆387–499)(no-tag)-R1, and pET15-SmaI-Mnn9-Van1(∆387–499)(no-tag)-F2, pET15-SmaI-Mnn9-Van1(∆387–499)(no-tag)-R2, respectively. The resulting DNA fragments were then inserted into the *Sma* I site of pET15-SmaI using the In-Fusion HD cloning kit, yielding pET15-SmaI-Mnn9-Van1(∆387–499)(no-tag).

### Determination of colony growth rate

The colony growth rates were measured as described previously (43). Briefly, conidia from each strain were point-inoculated into the center of MM plates. The colony diameters were measured after 24, 48, 72, 96, and 120 h of incubation at 37°C, and growth rates in mm/h were determined at each incubation interval (i.e., 24–48, 48–72, 72–96, and 96–120 h). The rates were averaged across the entire time interval. Measurements were performed 12 times for each strain.

### Analysis of conidiation efficiency

The conidiation efficiency was analyzed as described previously (44), with slight modification. Briefly, conidia from each strain were point-inoculated into the center of MM plates. After 5 days of incubation at 37°C, the conidia were suspended in 5  mL of 0.01% (wt/vol) Tween 20 and counted using a hemocytometer.

### Protein purification and quantification

Bacterial expression and purification of 6×His-tagged proteins were conducted following a previously described method (42). The yields of the recombinant proteins were 0.88 mg/L for Mnn2 and 1.25 mg/L for Mnn5.

### Enzyme assays

The synthetic acceptor substrate, *p*-nitrophenyl α-D-mannopyranoside (α-Man-pNP) or 4-methylumberlliferyl α-D-mannopyranoside (α-Man-4MU), was procured from Tokyo Chemical Industry Co., Ltd. (Japan). Standard assays were conducted with α-Man-pNP (1.5 mM) or α-Man-4MU (1.5 mM) as the acceptor, GDP-Man (10 mM) as the donor, and each purified protein (0.1 µg/µL) in a total reaction volume of 40 µL. The reaction mixture was incubated at 30°C for 16 h and terminated by heating at 99°C for 5 min. The reaction mixture was subjected to analysis by normal-phase HPLC using a Shodex Asahipak NH2P-50 4E amino column (250 × 4.6 mm^2^; Showa Denko, Japan), as previously detailed (42). Alpha-(1→2)-mannosidase was obtained by expressing the AfmsdS/AfmsdC gene in *E. coli* (45).

For enzymatic products, the supernatant underwent analysis by reverse phase-HPLC using an InertSustain C18 column (250 × 4.6 mm^2^; GL Science, Japan), as outlined previously (35). 4-methylumbelliferone derivatives were detected using UV_300_ absorbance. Jack bean mannosidase and α-(1→2, 3) Mannosidase were procured from New England Biolabs (USA).

### Preparation of total mannan fraction from *A. fumigatus*

The total mannan fractions from *A. fumigatus* were prepared as follows. The total mannan was extracted from *A. fumigatus* mycelia by autoclaving at 121°C for 2 h with 100 mM citrate buffer (pH 7.0) (6, 31, 42, 46). The cell wall extract was purified by cetyltrimethylammonium bromide (CTAB) using a previously described method (47), with slight modifications. CTAB (4 g) was dissolved in 150 mL of the cell wall extract and allowed to stand at 25°C for several hours. The solution was centrifuged at 12,000 × *g* for 15 min, and the supernatant was transferred to another tube. The precipitate was washed with 50 mL dH_2_O, followed by centrifugation at 12,000 × *g* for 15 min. The supernatant was combined with the previous supernatant. Boric acid was added at a concentration of 1% (wt/vol); while stirring, 2 M NaOH solution was added to adjust the pH to 8.8. The mixture was stirred well and stood for 1 h before centrifugation at 12,000 × *g* for 15 min. The precipitate was washed with 0.5% sodium acetate (pH 8.8) and dissolved in 50 mL of 2% acetic acid solution. Sodium acetate (1 g) and 150 mL of ethanol were added, followed by centrifugation at 12,000 × *g* for 15 min. The resultant sample was dialyzed in dH_2_O and lyophilized. To remove *O*-linked glycans, a β-elimination reaction was executed by subjecting the fractionated galactomannan to reduce alkali conditions (500 mM NaBH_4_/100 mM NaOH, 10 mL, at 25°C for 24 h). Following neutralization with a 50% acetic acid solution, the samples underwent overnight dialysis against distilled water. The purified samples were then lyophilized, resuspended in distilled water, and clarified using 0.45 µm pore filters. For the removal of galactofuran side chains, galactomannan underwent treatment with 100 mM hydrochloric acid at 100°C for 60 min (46). Subsequently, the samples were neutralized with 10 M NaOH and subjected to overnight dialysis against dH_2_O. The final product was defined as the total mannan fraction (46).

### Preparation of mannoproteins from *S. cerevisiae*

Mannoproteins were extracted from *S. cerevisiae* cells by autoclaving at 121°C for 2 h in 100 mM citrate buffer (pH 7.0) (6, 31, 42, 46). The extract was purified by CTAB using a previously described above.

### ^1^H-NMR analysis

^1^H-NMR analysis was performed following a previously established method (3, 31). In preparation for nuclear magnetic resonance spectroscopy, samples for NMR were exchanged twice in D_2_O with intervening lyophilization. They were then dissolved in D_2_O (99.97% atom 2H).

### Expression and detection of SucA

pPTR-II-SucA—a plasmid for expressing 3×FLAG-tagged SucA—was introduced into the *A. fumigatus* wild-type (A1151), Δ*mnn2*Δ*mnn5* (*hph*), ∆*mnn9,* and ∆*van1* strains (10), yielding A1151 +pPTR-II-SucA, ∆*mnn2*Δ*mnn5* + pPTR-II-SucA, ∆*mnn9* +pPTR-II-SucA, and ∆*van1* +pPTR-II-SucA, respectively. Each *A. fumigatus* mycelium was suspended in 1× glycoprotein denaturing buffer (0.5% SDS, 40 mM DTT) supplied with the Endo H product (New England Biolabs, Ipswich, MA, USA). Stainless steel beads were added to the samples, and the mycelia were crushed using a μT-12 bead cutter (TAITEC CORPORATION, Koshigaya, Japan). After crushing, samples were centrifuged at 12,000 × *g* for 10 min, and the supernatant was transferred to another tube as the cell wall protein fraction. In the case of Endo H treatment, GlycoBuffer 3 (50 mM sodium acetate, pH 6.0), which was supplied with the Endo H product, and Endo H were added to the cell wall protein fraction, and the mixture was incubated at 37°C for 10 h. Cell wall protein fractions were used as samples for SDS-PAGE by adding SDS sample buffer and boiling the mixture for 10 min. Western blotting was performed according to the established method (31), and a 5,000-fold dilution of monoclonal anti-FLAG antibody (clone M2; Merck KGaA, Darmstadt, Germany) was used as the antibody. EzWestLumiOne (ATTO CORPORATION, Tokyo, Japan) was used for chemiluminescence detection. The chemiluminescence imager used was MicroChemi (Berthold Technologies, Bad Wildbad, Germany).

## SUPPLEMENTAL MATERIAL

Supplemental material msphere.00100-24-s0001.pdf Fig. S1 to S4; Tables S1 and S2.

ASM does not own the copyrights to Supplemental Material that may be linked to, or accessed through, an article. The authors have granted ASM a non-exclusive, world-wide license to publish the Supplemental Material files. Please contact the corresponding author directly for reuse.
