# Supplementary material for: Discovery of α-(1→6)-linked mannan structures resembling yeast N-glycan outer chains in Aspergillus fumigatus mycelium
Source: mSphere. 2024 Apr 23;9(5):e00100-24. doi: 10.1128/msphere.00100-24 (PMC11237753; doi:10.1128/msphere.00100-24)
Supplement: Supplemental material — Fig. S1 to S4; Tables S1 and S2. [file msphere.00100-24-s0001.pdf]

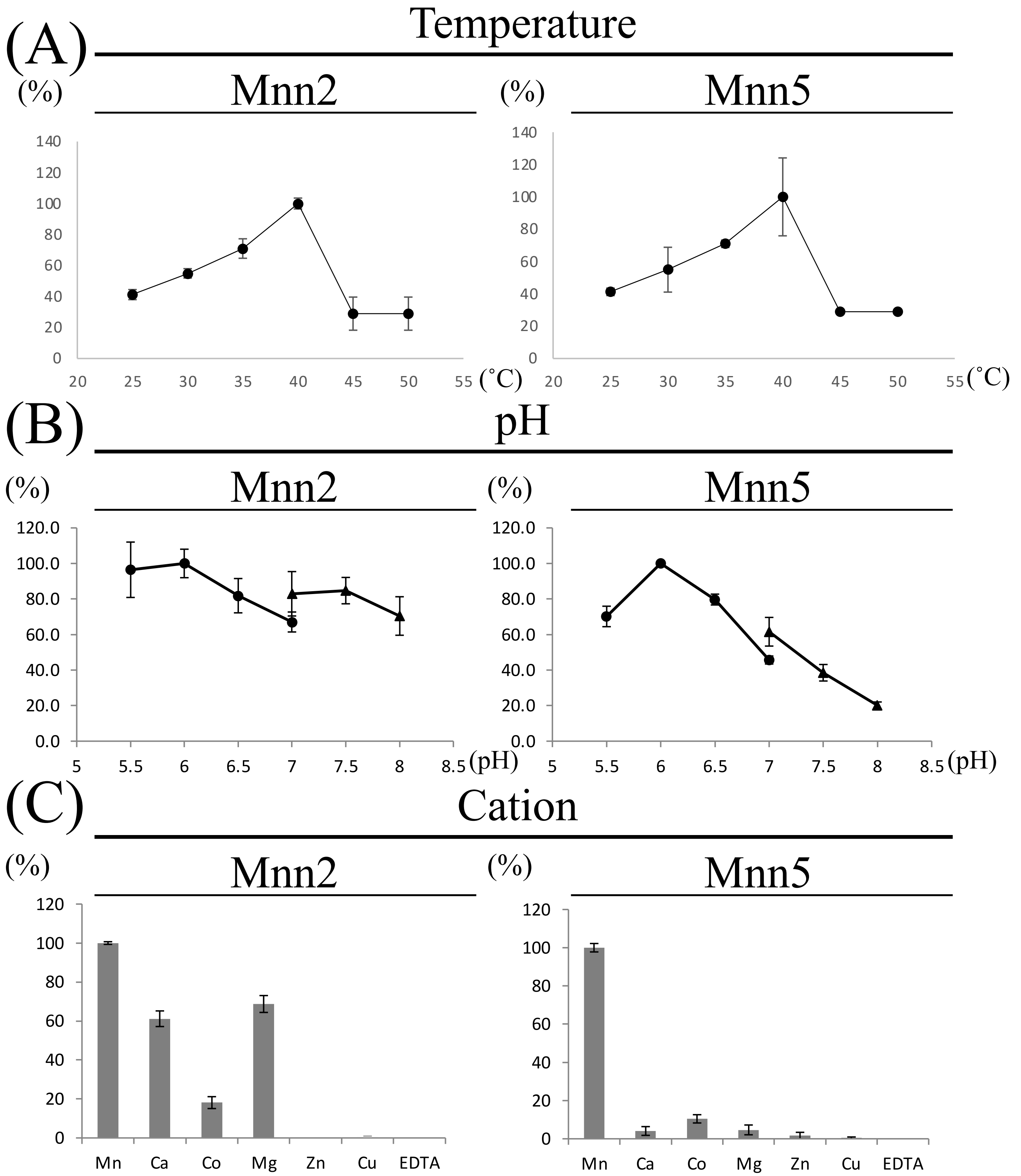

Fig. S1. Determination of the optimal temperature, pH range, and metal cation requirements for Mnn2 and Mnn5 activity. A reaction mixture (20 µL) containing 0.5 mM $MnCl_2$, 1.5 mM α-Man-pNP (substrate), 10 mM GDP-Man (donor), and 4.8 µg purified Mnn2 or Mnn5 was incubated for 18 h. (A) Effect of temperature on Mnn2 and Mnn5 enzymatic activity. A value of 100% corresponds to the incorporation of 1.11×10^{-4} nmol (α-Man-(1 → 2)-α-Man-pNP)/min/µg for Mnn2 and 2.58×10^{-4} nmol (α-Man-(1 → 2)-α-Man-pNP)/min/µg for Mnn5 at 40˚C. (B) Effect of pH on Mnn2 and Mnn5 enzymatic activity at 30˚C in 100 mM MES-NaOH (circles) or 100 mM MOPS-NaOH (triangles); 100% corresponds to the incorporation of 6.61×10^{-4} nmol (α-Man-(1 → 2)-α-Man-pNP)/min/µg for Mnn2 and 6.09×10^{-4} nmol (α-Man-(1 → 2)-α-Man-pNP)/min/µg for Mnn5 in 100 mM MES-NaOH at pH 6.0. (C) Metal cation requirements for Mnn2 and Mnn5 activity; reaction mixtures were incubated with 5 mM EDTA or various divalent metals (each 5 mM) at 30˚C. A value of 100% corresponds to the incorporation of 5.72×10^{-4} nmol (α-Man-(1 → 2)-α-Man-pNP)/min/µg for Mnn2 and 7.54×10^{-4} nmol (α-Man-(1 → 2)-α-Man-pNP)/min/µg for Mnn5 in 5 mM manganese. Data are presented as the mean ± SD (n = 3 independent experiments).

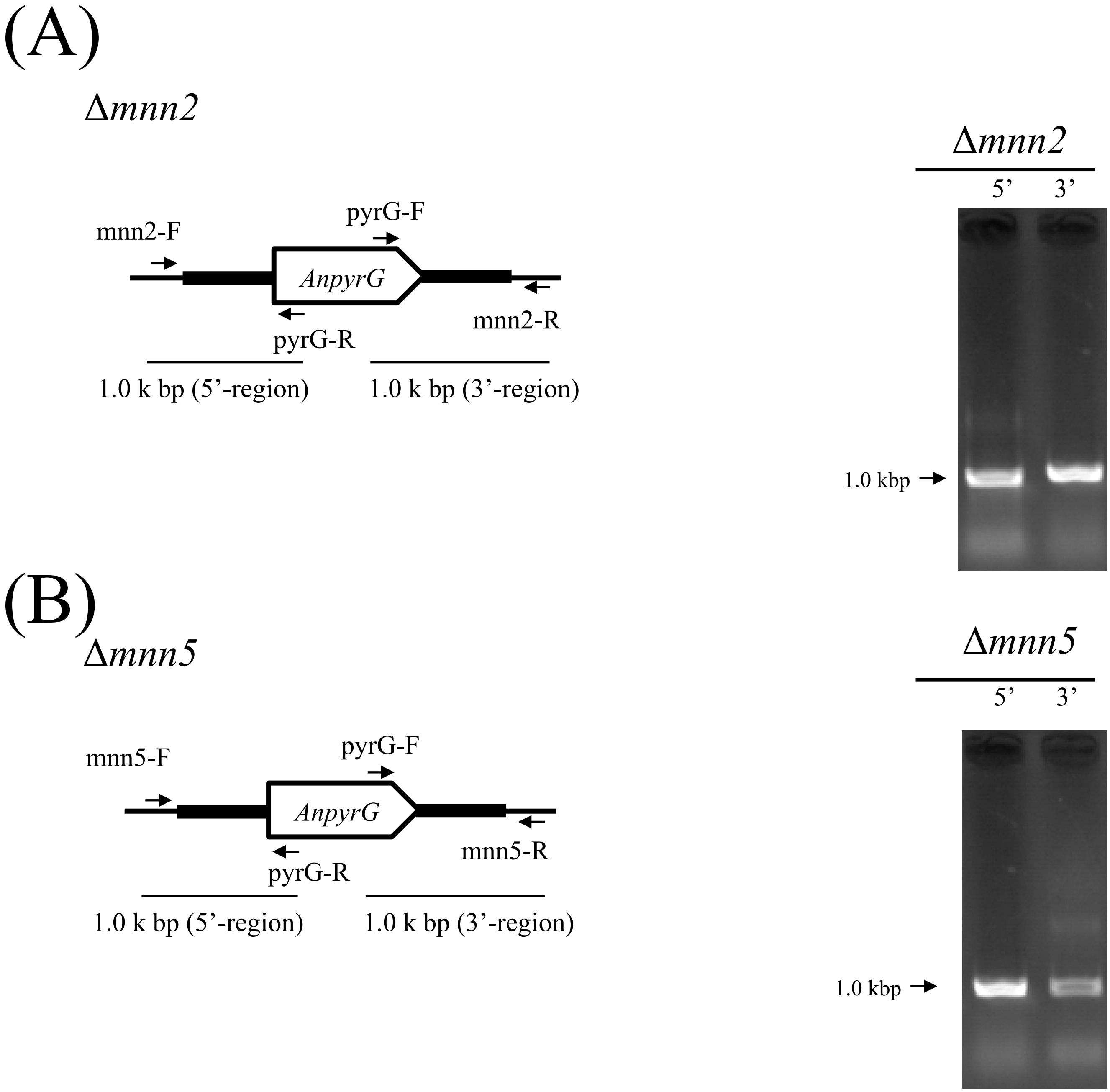

Fig. S2 Construction of the *A. fumigatus* strains. (A) Results of electrophoresis for PCR products when *mnn2* gene disruption via *AnpyrG* gene insertion was confirmed in the wild-type strain; (B) electrophoresis for PCR products when *mnn5* gene disruption via *AnpyrG* gene insertion was confirmed in the wild-type strain; (C and D) electrophoresis for PCR products when *mnn5* gene disruption via *ptrA* (C) or *hph* (D) gene insertion was confirmed in the Δ*mnn2* strain; (E–G) electrophoresis for PCR products when *mnn9* (E), *van1* (F), or *anpA* (G) gene disruption via *hph* gene insertion was confirmed in the Δ*mnn2*Δ*mnn5* strain

(C)

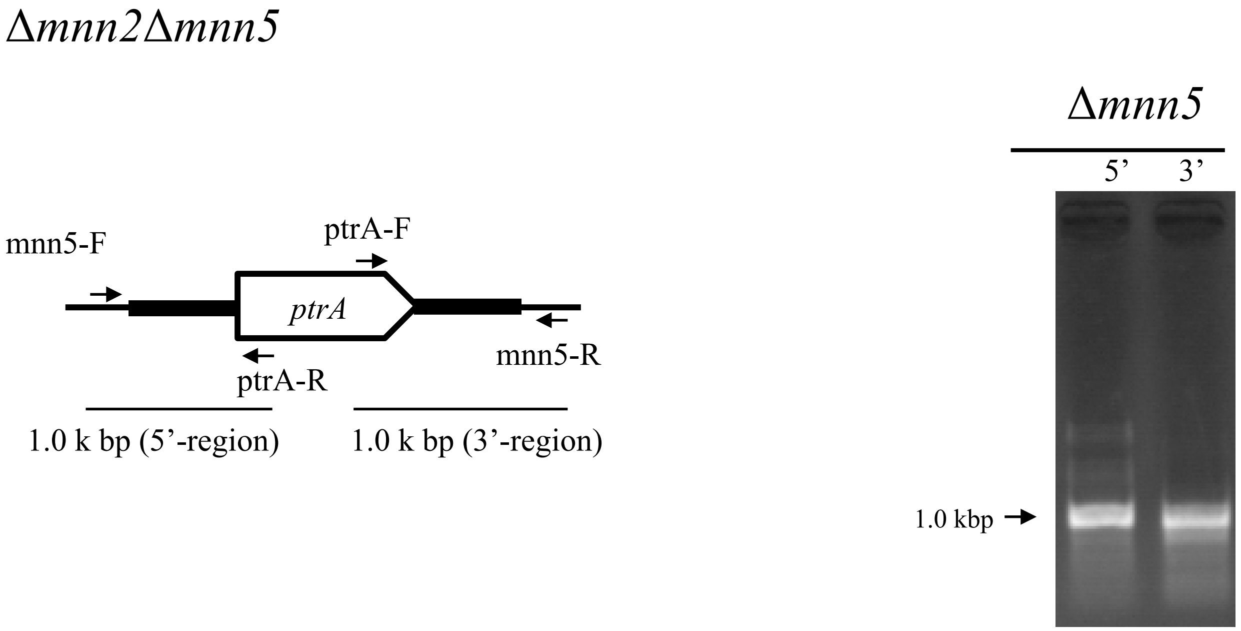

(D)

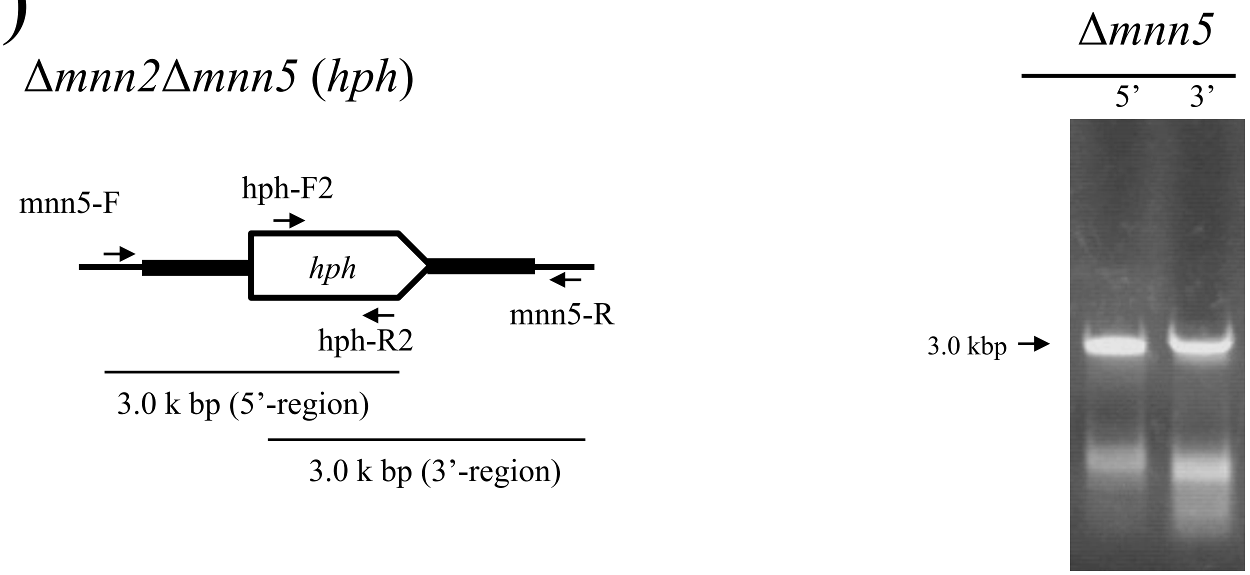

Fig. S2 continued

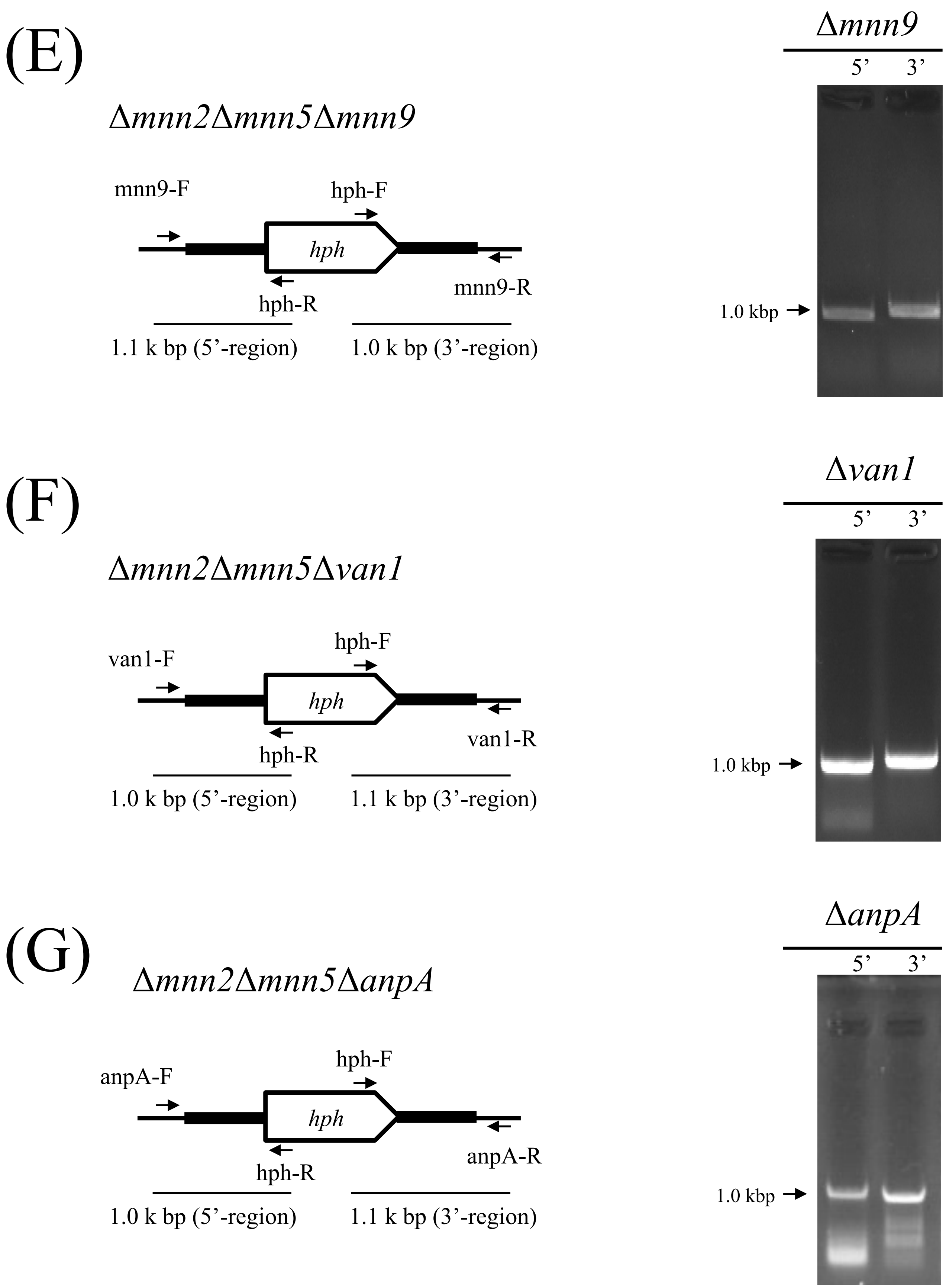

Fig. S2 continued

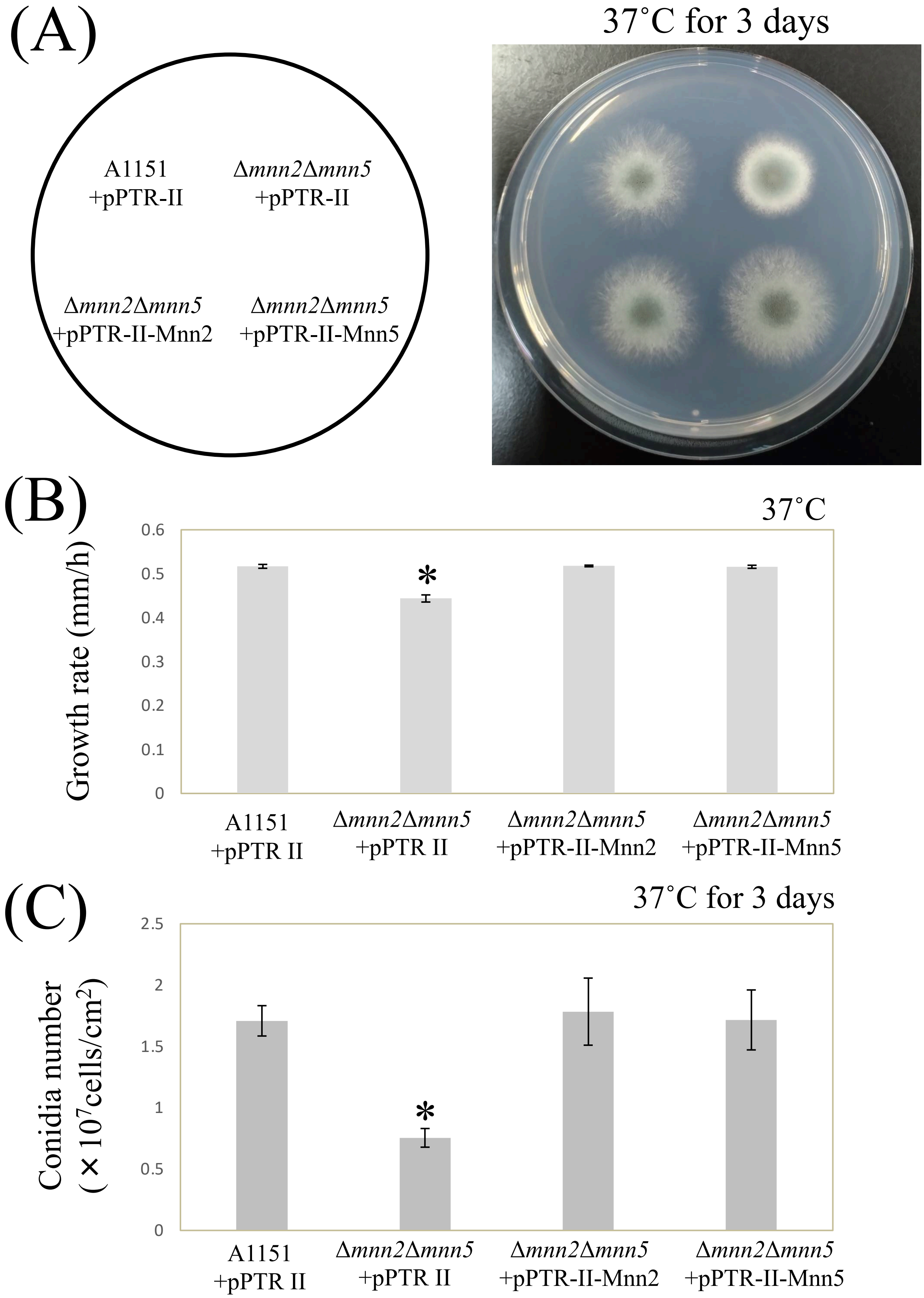

Fig. S3. Colony morphology of *A. fumigatus* A1151+pPTR-II, Δ*mnn2*Δ*mnn5*+pPTR-II, Δ*mnn2*Δ*mnn5*+pPTR-II-Mnn2, and Δ*mnn2*Δ*mnn5*+pPTR-II-Mnn5. (A) Colony morphology of wild-type (A1151)+pPTR-II, Δ*mnn2*Δ*mnn5*+pPTR-II, Δ*mnn2*Δ*mnn5*+pPTR-II-Mnn2, and Δ*mnn2*Δ*mnn5*+pPTR-II-Mnn5 on MM agar at 37° C for 3 days; the agar medium was inoculated with 1.0×10^4 conidiospores; (B) colony growth rates of wild-type (A1151)+pPTR-II, Δ*mnn2*Δ*mnn5*+pPTR-II, Δ*mnn2*Δ*mnn5*+pPTR-II-Mnn2, and Δ*mnn2*Δ*mnn5*+pPTR-II-Mnn5 on MM agar at 37° C; (C) conidia number per colony area of wild-type (A1151)+pPTR-II, Δ*mnn2*Δ*mnn5*+pPTR-II, Δ*mnn2*Δ*mnn5*+pPTR-II-Mnn2, and Δ*mnn2*Δ*mnn5*+pPTR-II-Mnn5 on MM agar at 37° C for 3 days; asterisks indicate significant differences (*, $p < 0.05$; Welch's *t*-test) from the results for the wild-type strain

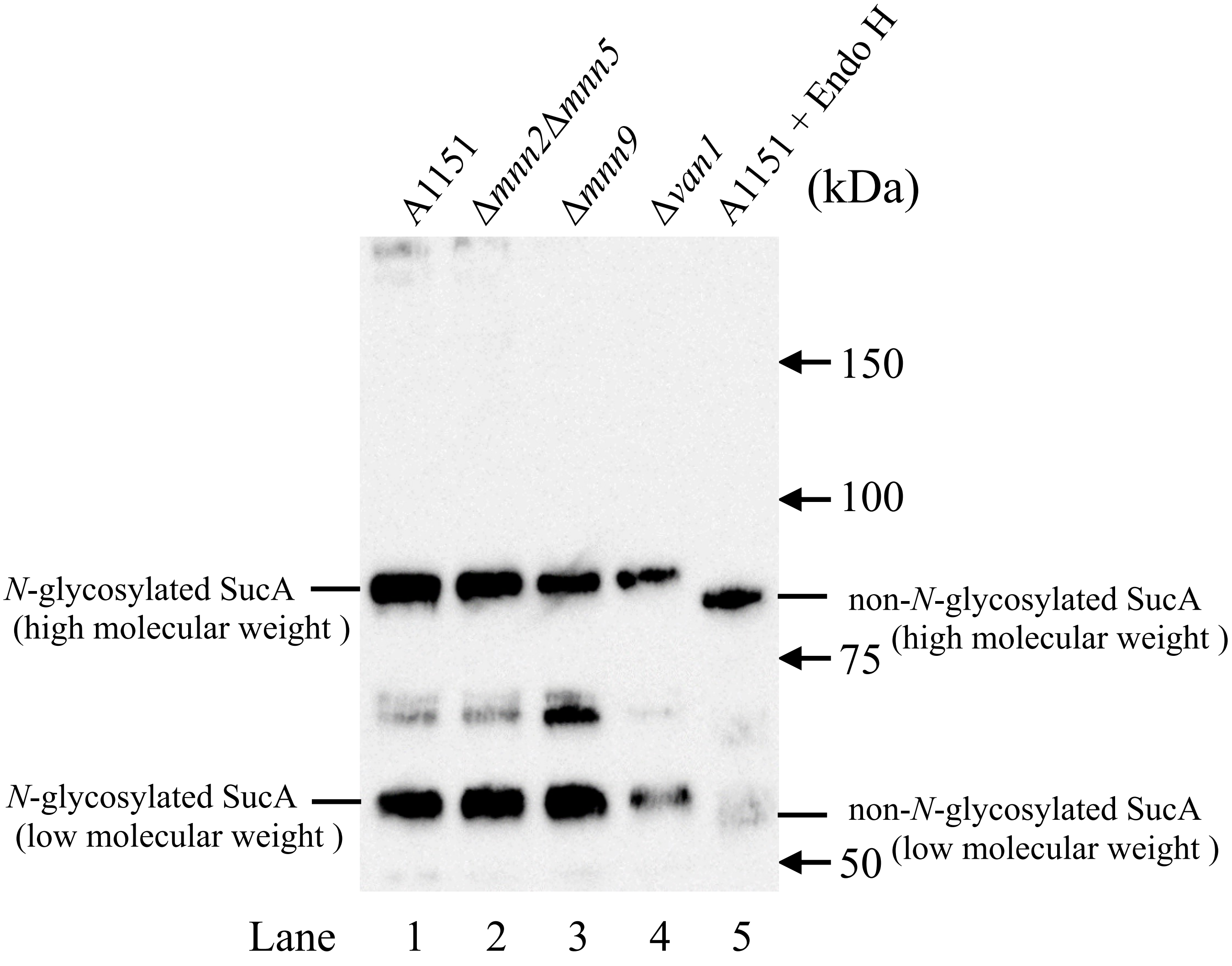

Fig. S4. Western blotting of *N*-glycosylated SucA of *A, fumigatus* A1151, Δ*mnn2*Δ*mnn5*, Δ*mnn9*, and Δ*van1*. Cells were grown, and proteins were extracted as described in the *Materials and Methods*; equal amounts of protein extracts were analyzed by 5%–20% SDS-PAGE and detected by chemiluminescence with anti-FALG antibody. Signals of SucA derived from A1151 (Lane 1), Δ*mnn2*Δ*mnn5* (Lane 2), Δ*mnn9* (Lane 3), Δ*van1* (Lane 4), and A1151 treated with Endo H (Lane 5)

Table S1. Strains used in the present study.

Strains	Genotype
Aspergillus fumigatus	
A1151	pyrG AF::Delta KU80
A1160	DeltaKU80 *pyrG -*
Δ*mnn2*	DeltaKU80 *pyrG - mnn2* Δ::*AnpyrG*
Δ*mnn5*	DeltaKU80 *pyrG - mnn5*Δ::*AnpyrG*
Δ*mnn2* Δ*mnn5*	DeltaKU80 *pyrG - mnn2*Δ ::*AnpyrG* , *mnn5*Δ ::*ptrA*
Δ*mnn2* Δ*mnn5Δmnn9*	DeltaKU80 *pyrG - mnn2*Δ ::*AnpyrG* , *mnn5*Δ ::*ptrA, mnn9*Δ*::hph*
Δ*mnn2* Δ*mnn5Δvan1*	DeltaKU80 *pyrG - mnn2*Δ ::*AnpyrG* , *mnn5*Δ ::*ptrA, van1*Δ*::hph*
Δ*mnn2* Δ*mnn5ΔanpA*	DeltaKU80 *pyrG - mnn2*Δ ::*AnpyrG* , *mnn5*Δ ::*ptrA, anpA*Δ*::hph*
Δ*mnn2* Δ*mnn5* (*hph*)	DeltaKU80 *pyrG - mnn2*Δ ::*AnpyrG* , *mnn5*Δ ::*hph*
Δ*mnn9*	DeltaKU80 *pyrG - mnn9*Δ ::*AnpyrG*
Δ*van1*	DeltaKU80 *pyrG - van1*Δ ::*AnpyrG*
A1151 + pPTR-II	A1151 harboring pPTR-II
Δ*mnn2* Δ*mnn5* + pPTR-II	Δ*mnn2* Δ*mnn5* (hph) harboring pPTR-II
Δ*mnn2* Δ*mnn5* + pPTR-II-Mnn2	Δ*mnn2* Δ*mnn5* (hph) harboring pPTR-II-Mnn2
Δ*mnn2* Δ*mnn5* + pPTR-II-Mnn5	Δ*mnn2* Δ*mnn5* (hph) harboring pPTR-II-Mnn5
A1151 + pPTR-II-SucA	A1151 harboring pPTR-II-SucA
Δ*mnn2* Δ*mnn5* + pPTR-II-SucA	Δ*mnn2* Δ*mnn5* (hph) harboring pPTR-II-SucA
Δ*mnn9* + pPTR-II-SucA	Δ*mnn9* harboring pPTR-II-SucA
Δ*van1* + pPTR-II-SucA	Δ*van1* harboring pPTR-II-SucA
Saccharomyces cerevisiae	
BY4741	*MAT* a *leu2Δ0 ura3Δ0 his3-Δ1 met15Δ0*
ΔSc*mnn5*	*MAT* a *leu2Δ0 ura3Δ0 his3-Δ1 met15Δ0 mnn5::kanMX4*
ΔSc*mnn2* Δ*Scmnn5*	*MAT* a *leu2Δ0 ura3Δ0 his3-Δ1 met15Δ0 mnn5::kanMX4 mnn2::HIS3*
ΔSc*mnn2* Δ*Scmnn5* + *Afmnn2*	Δ*Scmnn2* Δ*Scmnn5* harboring YEp352-GAP-AfMnn2
ΔSc*mnn2* Δ*Scmnn5* + *Afmnn5*	Δ*Scmnn2* Δ*Scmnn5* harboring YEp352-GAP-AfMnn5

Table S2. Oligonucleotide primers used in this study		
pHSG396-ptrA-IF-F	CGACTCTAGAGGATCGGGATCCCATTGGTAACGA	This study
pHSG396-ptrA-IF-R	CATCGATGGGGGATCCGATGAGCCGCTCTTGCAT	This study
pHSG396-F	CGAGGGGTCGACTCTAGAGG	Kadooka et al. 2022a
pHSG396-R	TACCCCATCGATGGGGGATC	Kadooka et al. 2022a
ScMnn2p-TM(1-26)-F	CATAAACAGAATTCGATGCTGCTTACCAAAAGGTTTTCAA	This study
ScMnn2p-TM(1-26)-R	ATGGTCTTTGTAGTCAATGACGAACAGCCCGCAC	This study
YEp352-GAPII-Mnn2-F	GATGACGATGACAAGCGATCTGTTTCAGTCGAGCCA	This study
YEp352-GAPII-Mnn2-R	GATCCCCGGGTACCGTCACGTCTTGACCGACTGC	This study
ScMnn5p-TM(1-35)-F	CATAAACAGAATTCGATGCTGATTAGGTTAAAGAAGAGAA	This study
ScMnn5p-TM(1-35)-R	ATGGTCTTTGTAGTCCCAACTAGAAGACACATCATTATG	This study
YEp352-GAPII-Mnn5-F	GATGACGATGACAAGACCCCAGCACCAGCATCC	This study
YEp352-GAPII-Mnn5-R	GATCCCCGGGTACCGTCAATCATCGGTAAACTTTGGATCG	This study
3xFALG-F	GACTACAAAGACCATGACGGTGA	This study
3xFALG-R	CTTGTCATCGTCATCCTTGTAA	This study
mnn2-1	ATCATCACTCGACGCAGACG	This study
mnn2-2	AGAGTCGACCCCTCGAAAGACGAGCGTGAGCAGTC	This study
mnn2-3	CCCATCGATGGGGTAGAACGGGTGTCACTGTGAAG	This study
mnn2-4	GTCCGCTCAGCCGTACTTAA	This study
mnn5-1	CAACTCACATAGGCAGGCAC	This study
mnn5-2	AGAGTCGACCCCTCGGGCTGCGTTTGAAGGGAAAT	This study
mnn5-3	CCCATCGATGGGGTAAGCCCAAGATTCTCAAAGGC	This study
mnn5-4	CTGGTAGCTCATACGCTGGT	This study
mnn9-1	GGCCGGGGGTATATCAGCT	Kadooka et al. 2022b
mnn9-2	AGAGTCGACCCCTCGGACGACCGGTGAAAACGAGC	Kadooka et al. 2022b
mnn9-3	CCCATCGATGGGGTAGCGGCTAGGGTACTCTGTTTA	Kadooka et al. 2022b
mnn9-4	TCACCCACAACATCACCGAC	Kadooka et al. 2022b
van1-1	CTTGGACAGCGACTACGAGG	Kadooka et al. 2022b
van1-2	AGAGTCGACCCCTCGCGGGGGTAGGCAAAGTAGC	Kadooka et al. 2022b
van1-3	CCCATCGATGGGGTAGTGACAGGGACAGCAGAGGT	Kadooka et al. 2022b
van1-4	GTGACGCGTTAGGCATTTCCT	Kadooka et al. 2022b
anpA-1	CGCTCCGAACTGCATGCAAT	Kadooka et al. 2022b
anpA-2	AGAGTCGACCCCTCGTATCAAGAATCGCGGCCGTG	Kadooka et al. 2022b
anpA-3	CCCATCGATGGGGTAAACCAGGCTGAAACGGAAGG	Kadooka et al. 2022b
anpA-4	ACGCGATCTACTTCAATTGCCC	Kadooka et al. 2022b
mnn2-F	CTGCGGTTGCTGATGCATG	This study
mnn2-R	TCAGGTGCAATTCTAGCACG	This study
mnn5-F	TAACAGCCCAAGGTAACGCA	This study
mnn5-R	CCACTAATTGACCTCACGCAA	This study
mnn9-F	CGATGTCGGCGCCAGTAATT	Kadooka et al. 2022b
mnn9-R	GTCAAACTAGGCGGCTACCTC	Kadooka et al. 2022b
van1-F	GAAACAGTGGAGCTCTGGGG	Kadooka et al. 2022b
van1-R	GGTAGGTTCGGTTTGGAGTGC	Kadooka et al. 2022b
anpA-F	TAAGCAGCTGTACACACCGG	Kadooka et al. 2022b
anpA-R	GGCAAAAGGACCGTGTCACA	Kadooka et al. 2022b
pET15-SmaI-F	CTCTTTCAGGGACCCGGGTACCAGGATCCGGCTGCTAACAAAGCCCGAAAG	This study
pET15-SmaI-R	GGGTCCCTGAAAGAGGCCGCTGCTGTGATGATG	This study
pET15b-Mnn2-F	ACAGCAGCGGCCATAGCCGATCTGTTTCAGTCGAGCCA	This study
pET15b-Mnn2-R	CACTCGAGTGCGGCCTCACGTCTTGACCGACTGC	This study
pET15b-Mnn5-F	ACAGCAGCGGCCATAGCACCCCAGCACCAGCATCC	This study
pET15b-Mnn5-R	CACTCGAGTGCGGCCTCAATCATCGGTAAACTTTGGATCG	This study
pET15b-Mnn9-F	ACAGCAGCGGCCATAGCTCTGCCTCCCCCGTTAATGC	This study
pET15b-Mnn9-R	CACTCGAGTGCGGCCTTATTCGTTCTGATGATATACAAAATAA	This study
gBlocks-Van1	CTCTTTCAGGGACCCCTGCCTACCCTGTCTCTTGGTCTGATCCACTCATCAGAGGATCTTCAGCTGCAAACCGTGCGCTACTATGATCTGTCCA<br>AAGTTCAGGGAACGGCGAAAGGCTGGGAGAAAGGTGAACGGGTCTTAATGTTGACCCCGTTGCGTGATGCCTCTAGCCATCTGCCGATGTT<br>CTTTAGCCATCTCCGGAATCTGACCTATCCGCACAACTTAATTGACCTGGCTTTTCTCGTTAGCGACTCGAAAGACGATACGTTAGGGATGCT<br>GACACGCATGCTGGAGGAAGTCCAAAACGATCCAGACCCGAAAATGACCTTTGGAGAAATCAGCGTAATCCAGAAGGATTTCGGTCAGAA<br>AGTGCAGCAGGACGTAGAGAGTCGCCATGGGTTTGCCGCCCAAGCAGGCCGCCGCAAACTGATGGCTCAGGCCCGTAACTGGCTGCTGTCC<br>GCAACTTTGCGTCCAACACACTCGTGGGTCTATTGGCGTGATGCGGACGTGGAAACCGCGCCTCGCACCATTCTGGAGGACCTGATGCGCCA<br>TGATAAAGACGTGATTGTGCCGAATGTTTGGCGTCCACTCCCGGATTGGCTCGGCGGAGAGCAGCCGTACGATCTGAACAGTTGGCAGGAA<br>AGCGAAACTGCGCTGGCGTTAGCGGAAACTCTGGATGAAGATGCGGTAATTGTCGAAGGCTATGCGGAATACGCAACTTGGCGTCCTCACT<br>TGGCGTATCTTCGTGATCCCTTTGGTGATCCGGACATGGAAATGGAACTGGACGGTGTTGGTGGCGTGTCCATCCTTGCGAAAGCCAAAGTT<br>TTCCGCAGTGGCGTTCACTTTCCGGCTTTCTCGTTCGAAAAGCATGCGGAAACGGAGGCGTTTGGCAAAATGGCCAAACGCATGGGGTTTTC<br>GGTGATTGGGTTGCCCCACTACACGATTTGGCATCTGTATGAGCCGTCAGTAGATGACTTACGCCATATGGAGGAGATGGAGCAAGAACGT<br>AAAGCACGCGAACGCGAAGAAAAGGAACAAGCCGAACGTGCTGAACGCATGAAAGCACTGTTCGTAGAACCGGGTCCGCAATGGGACATC<br>GATAAAGCGTTTGTGCAGGATAGCATTCAGACGGAAAAGGAAGGCCCCGATCAAACGAAAGCGGATGGTGTCACAGGCACCGCTGAGGTG<br>AAAGATGTGCAGGGCAGTGCTAGCCATCCTGTCGCCGCCTCTCCCGAACCATCCGTTAATGCCGCACCAGTGGCACCGGTTGCACGTGGTGG<br>CCCGGGTGAAACCGAACGCAAGCTGTAAGGGTACCAGGATCCG	This study
pET15-SmaI-Van1(Δ387-499)-F	CTCTTTCAGGGACCCCTGC	This study
pET15-SmaI-Van1(Δ387-499)-R	CGGATCCTGGTACCCTTATGACGGCTCATACAGATGCC	This study
pET15-SmaI-Mnn9-Van1(no-tag)-F1	CTCTTTCAGGGACCCTCTGCCTCCCCCGTTAATGC	This study
pET15-SmaI-Mnn9-Van1(no-tag)-R1	AGTTAAACAAAATTATTATTCGTTCTGATGATATACAAAATAATCTGGC	This study
pET15-SmaI-Mnn9-Van1(no-tag)-F2	TAATTTTGTTTAACTTTAAGAAGGAGATATACCATGCTGCCTACCCTGTCTCTTGGT	This study
pET15-SmaI-Mnn9-Van1(no-tag)-R2	CGGATCCTGGTACCCTTATGACGGCTCATACAGATGCC	This study
hph-F	CCGCGGGATCCACTTAACG	This study
hph-R	GTCTCTCCGCATGCCAGAAA	This study
hph-F2	GACGAACTCGTGAGCTCTGTAC	This study
hph-R2	CACTAGAAGGCACTCTTTGCTG	This study
pyrG-F	GATCTACCCCTTGGAACGCA	This study
pyrG-R	GACCATCGTGGGCAATTGGT	This study
ptrA-F	CATATGTAAATGGCTGTGTCCCG	This study
ptrA-R	TTTAGCTTTGACCGGTGAGC	This study
ScMNN2-HIS3-F	ATGCTGCTTACCAAAAGGTTTTCAAAGCTGTTCAAGCTGACGTTCATAGTCTCTTGGCCTCCTCTAGTACAC	This study
ScMNN2-HIS3-R	TTATTTACCGGCAATCGCCTTATCATGAGTAGATTCTAAAAATGCTAATCCGCCTCGTTCAGAATGACAC	This study
ScMNN2-F	TGAGGTGGAATGAGCGATGG	This study
ScMNN2-R	TTTCGTTGACGCTAGCATTCG	This study
HIS3-F	TCCCTCCACCAAAGGTGTTC	This study
HIS3-R	AAAAGAGTCATCCGCTAGGTGG	This study
pPTR-II-MnnA-F	CTCTAGAGGATCCCCTGCTCTGCGGTTGCTGATG	This study
pPTR-II-MnnA-R	TCGAGCTCGGTACCCGCTGGGGTGTATATGACCTGC	This study
pPTR-II-MnnB-F	CTCTAGAGGATCCCCAGAAACAGCAGCTAACAGCCC	This study
pPTR-II-MnnB-R	TCGAGCTCGGTACCCAATCGACAAGGAGCTGCAGG	This study
